# GATA2 controls lymphatic endothelial cell junctional integrity and lymphovenous valve morphogenesis through *miR-126*

**DOI:** 10.1242/dev.184218

**Published:** 2019-11-01

**Authors:** Md. Riaj Mahamud, Xin Geng, Yen-Chun Ho, Boksik Cha, Yuenhee Kim, Jing Ma, Lijuan Chen, Greggory Myers, Sally Camper, Debbie Mustacich, Marlys Witte, Dongwon Choi, Young-Kwon Hong, Hong Chen, Gaurav Varshney, James Douglas Engel, Shusheng Wang, Tae-Hoon Kim, Kim-Chew Lim, R. Sathish Srinivasan

**Affiliations:** 1 Cardiovascular Biology Research Program, Oklahoma Medical Research Foundation, Oklahoma City, OK 73104, USA; 2 Department of Cell Biology, University of Oklahoma Health Sciences Center, Oklahoma City, OK 73117, USA; 3 Department of Biological Sciences and Center for Systems Biology, The University of Texas at Dallas, Richardson, TX 75080, USA; 4 Department of Cell and Molecular Biology, Tulane University, New Orleans, LA 70118, USA; 5 Department of Cell and Developmental Biology, University of Michigan Medical School, Ann Arbor, MI 48109, USA; 6 Department of Surgery, University of Arizona, Tuscon, AZ 85724, USA; 7 Department of Surgery, Keck School of Medicine, University of Southern California, Los Angeles, CA 90033, USA; 8 Vascular Biology Program, Boston Children's Hospital, Boston, MA 02115, USA; 9 Genes & Human Disease Research Program, Oklahoma Medical Research Foundation, Oklahoma City, OK 73104, USA

**Keywords:** Lymphatic vasculature, Lymphovenous valves, GATA2, miR-126, Claudin 5, VE-cadherin

## Abstract

Mutations in the transcription factor GATA2 cause lymphedema. GATA2 is necessary for the development of lymphatic valves and lymphovenous valves, and for the patterning of lymphatic vessels. Here, we report that GATA2 is not necessary for valvular endothelial cell (VEC) differentiation. Instead, GATA2 is required for VEC maintenance and morphogenesis. GATA2 is also necessary for the expression of the cell junction molecules VE-cadherin and claudin 5 in lymphatic vessels. We identified *miR-126* as a target of GATA2, and *miR-126^−/−^* embryos recapitulate the phenotypes of mice lacking GATA2. Primary human lymphatic endothelial cells (HLECs) lacking GATA2 (HLEC^ΔGATA2^) have altered expression of claudin 5 and VE-cadherin, and blocking *miR-126* activity in HLECs phenocopies these changes in expression. Importantly, overexpression of *miR-126* in HLEC^ΔGATA2^ significantly rescues the cell junction defects. Thus, our work defines a new mechanism of GATA2 activity and uncovers *miR-126* as a novel regulator of mammalian lymphatic vascular development.

## Abstract (precis)

**Summary:** GATA2 and its target gene *miR-126* are novel regulators of lymphatic endothelial cell junctions, which are important regulators of vascular morphogenesis.

## INTRODUCTION

The lymphatic vasculature is a hierarchically organized tissue that absorbs and returns extravasated plasma fluids and digested lipids to the blood circulation (Chen et al., 2014; Tammela and Alitalo, 2010). This fluid, commonly known as lymph, is absorbed by lymphatic capillaries and transported via collecting lymphatic vessels. Lymphatic valves (LVs) within the lymphatic vessels regulate the unidirectional flow of lymph. Finally, lymph is returned to the blood circulation at the junction of jugular and subclavian veins through four lymphovenous valves (LVVs).

Mutations in multiple genes are associated with lymphedema, a debilitating disease characterized by the swelling of tissues, most obviously the limbs (Brouillard et al., 2014). Other lymphatic anomalies include chylous ascites (fluid in the peritoneal cavity), chylothorax (fluid around the lungs) or lymph reflex. These lymphatic vascular dysfunctions could arise from anatomical defects in the vessels or valves, though in most cases the precise cause is unclear.

Heterozygous mutations in the zinc-finger transcription factor *GATA2* are associated with an array of hematopoietic disorders and lymphedema (Spinner et al., 2014). The overlapping phenotypes of these diseases include immune deficiency, myelodysplasia (MDS), acute myeloid leukemia (AML), predisposition to mycobacterial infections and warts, hearing loss and lymphedema (Crispino and Horwitz, 2017; Spinner et al., 2014). Emberger syndrome, caused by mutations in *GATA2*, is classified as deafness and primary lymphedema with MDS/AML (Emberger et al., 1979; Kazenwadel et al., 2012; Ostergaard et al., 2011). Approximately 11-30% of people with mutated *GATA2* develop lymphedema (Donadieu et al., 2018; Kazenwadel et al., 2012; Ostergaard et al., 2011; Spinner et al., 2014). Donadieu et al. noted that individuals with *GATA2* mutations tend to develop lymphedema early, in the first decade of life. In summary, early-onset lymphedema with incomplete penetrance is associated with *GATA2*-heterozygous mutations. We need better insight into the molecular mechanisms of GATA2 activity to understand the causes of lymphedema in Emberger syndrome patients.

Mouse models have revealed that GATA2 is crucial for the development of a variety of cell types, including hematopoietic cells, neurons, pituitary gland cells, urinogenital system cells and endothelial cells (Charles et al., 2006; Craven et al., 2004; Khandekar et al., 2007; Lim et al., 2012; Zhou et al., 1998, 2000). *Gata2^−/−^* mice die at embryonic day (E)10 just as lymphatic endothelial cells (LECs) are starting to be specified. Conditional deletion of *Gata2* from all endothelial cells during mouse development results in severely edematous embryos with small blood-filled lymph sacs (Frye et al., 2018; Lim et al., 2012). Conditional deletion of *Gata2* in LECs results in mispatterned dermal lymphatic vessels, and a loss of LVs (Frye et al., 2018; Kazenwadel et al., 2015). In addition, E12.5 or older embryos with a conditional deletion of *Gata2* in all endothelial cells or LECs lack LVVs (Frye et al., 2018; Geng et al., 2016; Kazenwadel et al., 2015). Thus, GATA2 is essential for proper development of the lymphatic vasculature.

*In vitro* experiments have revealed several molecular functions of GATA2. A stiff extracellular matrix (ECM) triggers GATA2-dependent activation of *VEGFR2* (*KDR*) expression in blood endothelial cells (Mammoto et al., 2009). In contrast, a soft ECM enhances *GATA2* expression in primary human LECs (HLECs) and, in turn, induces *VEGFR3* (*FLT4*) (Frye et al., 2018). This mechanistic relationship has been proposed to be crucial for LEC migration from the cardinal vein and could explain the small lymph sacs in mice lacking *Gata2* in all endothelial cells.

Oscillatory shear stress (OSS), Wnt/β-catenin signaling and PROX1 are thought to be the most-upstream regulators of LV and LVV formation, all of which activate *GATA2* expression in HLECs (Cha et al., 2016, 2018; Kazenwadel et al., 2015; Sweet et al., 2015). OSS-induced GATA2 expression in HLECs is dependent on histone deacetylase 3 (HDAC3) (Janardhan et al., 2017). In turn, GATA2 is necessary for OSS-induced expression of FOXC2 and connexin 37 (GJA4) (Kazenwadel et al., 2015; Sweet et al., 2015). Furthermore, GATA2 directly associates with the regulatory elements of PROX1 in HLECs, and GATA2 knockdown in HLECs downregulates the expression of PROX1 (Kazenwadel et al., 2015).

The current model built on these observations proposes that GATA2 regulates the differentiation of valvular endothelial cells from progenitors by upregulating PROX1, FOXC2 and connexin 37 in those cells. However, whether this model is accurate *in vivo* remains unclear. Although LVV-forming endothelial cells (LVV-ECs) differentiate at E12.0 with the upregulation of PROX1, FOXC2, connexin 37 and GATA2 in those cells (Geng et al., 2016), whether GATA2 is necessary for LVV-EC differentiation is not known. To address these questions, we investigated the role of GATA2 during LVV-EC differentiation and performed unbiased RNA-seq analysis to identify the physiologically significant targets of GATA2.

## RESULTS

### GATA2 is necessary for the proper architecture of newly differentiated LVV-ECs

**Fig. 1.**
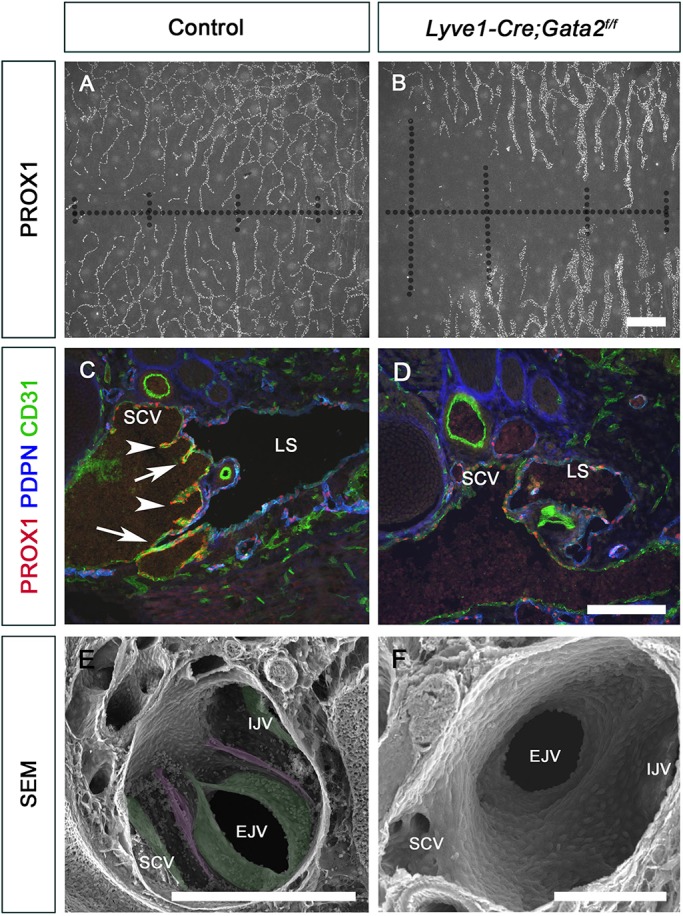
**Lymphatic vessels are defective and LVVs are absent in *Lyve-Cre;Gata2^f/f^* embryos.** E16.5 control and *Lyve-Cre;Gata2^f/f^* littermates were analyzed. (A,B) The lymphatic vessels in the dorsal skin of mutants were hypoplastic, dilated and had fewer branch points. Dotted lines indicate the dorsal midline of the skin. (C,D) LVVs (arrows) and VVs (arrowheads) were seen in control (C), but not in mutants (D). (E,F) SEM confirmed that LVVs (magenta) and VVs (green) were present in control (E) but not in mutant (F) embryos. EJV, external jugular vein; IJV, internal jugular vein; LS, lymph sac; SCV, subclavian vein. (A,B) *n*=3 embryos; (C,D) *n*=3 embryos and 6 LVVs per genotype; (E,F) *n*=3 embryos and 5 LVV complexes per genotype. Scale bars: 500 μm (A,B); 200 μm (C,D); 300 μm (E); 100 μm (F).

Previous reports, including ours, have used pan-endothelial Cre lines for deleting *Gata2* (Frye et al., 2018; Geng et al., 2016; Kazenwadel et al., 2015). *Gata2* has also been deleted in the lymphatic vasculature in a mosaic manner using tamoxifen-inducible Cre lines (Frye et al., 2018; Kazenwadel et al., 2015). Here, we used *Lyve1-Cre* (Pham et al., 2010) to delete *Gata2* (Charles et al., 2006) in the lymphatic vasculature. Using lineage tracing we have determined that *Lyve1-Cre* efficiently and constitutively labels LECs from E11.5 (data not shown). *Lyve1-Cre* is also expressed in a subset of blood endothelial cells and leukocytes (Dellinger et al., 2013; Takeda et al., 2016). As anticipated, *Lyve1-Cre;Gata2^f/f^* (*Gata2^LECKO^*) embryos recapitulated the previously reported lymphatic vascular phenotypes. Specifically, E16.5 *Gata2^LECKO^* embryos possessed blood-filled lymphatic vessels, which were dilated and had fewer branch points. The mutant embryos also lacked LVs and LVVs (Fig. 1; data not shown).

**Fig. 2.**
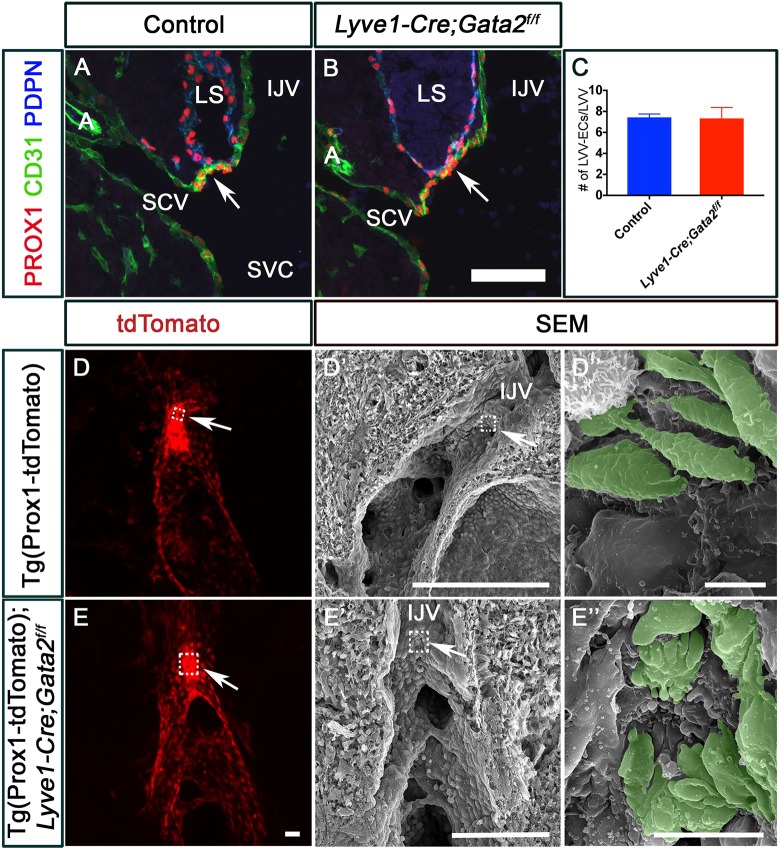
**GATA2 is required for the proper architecture of lymphovenous valve-forming endothelial cells** **(LVV-ECs).** (A-C) PROX1^high^ LVV-ECs (arrows) were observed in both E12.0 control (A) and *Lyve1-Cre;Gata2^f/f^* (B) embryos. Blood cells were seen within the lymph sacs of mutant embryos. (C) No obvious difference in LVV-EC numbers was observed between the two genotypes. (D-E″) E12.0 Tg(Prox1-tdTomato) (D) and Tg(Prox1-tdTomato); *Lyve1-Cre;Gata2^f/f^* (E) embryos were sagittally sectioned along the internal jugular vein. The fluorescent signal from the reporter revealed LVV-ECs in both control and mutant embryos (arrows). (D′,D″,E′,E″) The samples from D and E were analyzed using SEM, which revealed the LVV-ECs (pseudocolored in green) with elongated morphology in control embryos (D′ and magnified figure of the boxed region in D″). In contrast, the LVV-ECs of mutant embryos were dysplastic (E′ and magnified figure of the boxed region E″). A, artery; IJV, internal jugular vein; LS, lymph sac; SCV, subclavian vein; SVC, superior vena cava. (A-E) *n*=3 embryos and 6 LVV complexes per genotype per stage. Scale bars: 100 μm (A,B,E′); 50 μm (D,E); 200 μm (D′); 5 μm (D″); 10 μm (E″).

To investigate a potential role for GATA2 in LVV-EC differentiation, we used numerous LVV-EC markers (PROX1^high^, FOXC2^high^, connexin 37, integrin-α5, integrin-α9) to analyze E12.0 embryos (Fig 2A,B, Fig. S1A-H). LVV-EC numbers were not significantly different between control and *Gata2^LECKO^* littermates at this stage (Fig. 2C). We also analyzed E12.0 Tie2-Cre*;Gata2^f/f^* embryos, in which *Gata2* is deleted from all endothelial cells at a much earlier time point (Kisanuki et al., 2001). E12.0 Tie2-Cre*;Gata2^f/f^* embryos had LVV-ECs (Fig. S2), excluding the possibility that the LVV-ECs observed in *Gata2^LECKO^* embryos are the result of inefficient deletion of *Gata2* by *Lyve1-Cre.* Thus, LVV-EC differentiation is normal in *Gata2^LECKO^* mutants.

To examine LVV-ECs further, we used correlative fluorescence microscopy followed by scanning electron microscopy (SEM) to visualize the developing LVV-ECs at high resolution (Geng et al., 2016). First, we analyzed sagittal sections along the cardinal vein of E12.0 Tg(Prox1-tdTomato) embryos by confocal microscopy (Gong et al., 2003). We observed tdTomato^high^ LVV-ECs in both control and *Gata2^LECKO^* backgrounds (Fig. 2D,E). SEM on these same samples (Fig. 2D′,E′) revealed individual LVV-ECs that are elongated and aligned perpendicular to the direction of blood flow in control E12.0 embryos (Fig. 2D″, pseudocolored in green). The rest of the venous endothelium was quiescent with cobblestone-like appearance. In contrast, SEM revealed that the LVV-ECs in E12.0 Tg(Prox1-tdTomato);*Gata2^LECKO^* embryos are round and not aligned perpendicular to blood flow (Fig. S3). In addition, LVV-ECs also appear dysplastic in E12.0 Tg(Prox1-tdTomato);*Gata2^LECKO^* embryos (Fig. 2E″, pseudocolored in green; Fig. S3). Based on these observations, we conclude that GATA2 is not necessary for the differentiation of LVV-ECs or for the upregulation of PROX1 or FOXC2 in those cells. However, GATA2 is necessary for the proper architecture of the newly formed LVV-ECs.

### GATA2 is necessary for the maintenance and morphogenesis of LVV-ECs

**Fig. 3.**
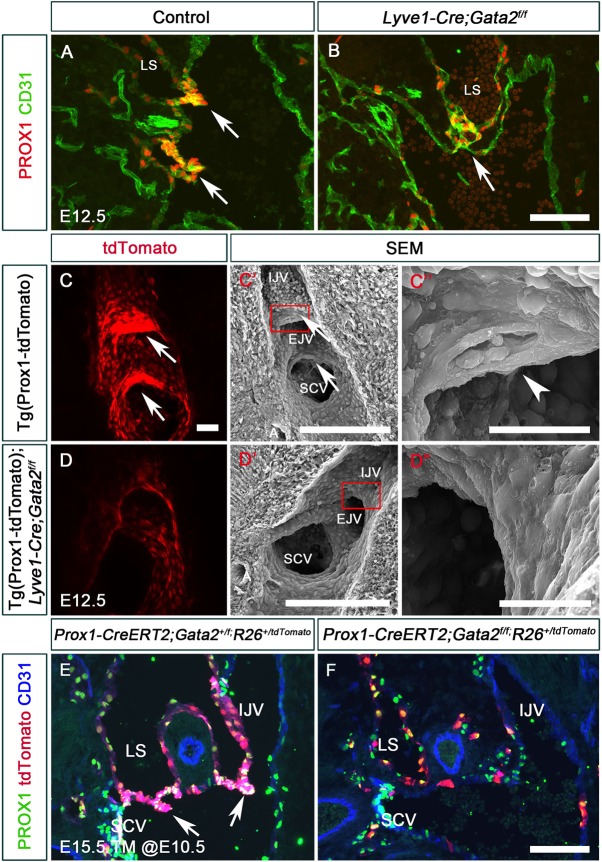
**LVV-ECs are lost from E12.5 embryos lacking GATA2.** (A,B) E12.5 wild-type and *Lyve1-Cre;Gata2^f/f^* embryos were analyzed by immunohistochemistry on sections. LVV-ECs had invaginated into the vein in control embryos (A, arrows). In contrast, very few PROX1^+^ cells were observed in mutant embryos (B, arrow). (C,D) E12.5 Tg(Prox1-tdTomato) and Tg(Prox1-tdTomato); *Lyve1-Cre;Gata2^f/f^* embryos were sagittally sectioned along the internal jugular vein and the fluorescent signal was analyzed using confocal microscopy. LVVs with strong tdTomato signal were seen in control (C, arrows), but not in mutant (D) embryos. (C′,C″,D′,D″) The samples from C and D were re-processed and analyzed by SEM. In controls, LVV-ECs (arrows) with elongated morphology were observed at the junction of the internal and external jugular veins and at the junction of the internal jugular vein and the subclavian vein. An opening connecting the lymph and blood circulations was also seen (arrowhead in C″). In contrast, endothelial cells at the junction of veins were indistinguishable from the rest of the venous endothelial cells in mutant embryos (D′,D″). C″ and D″ are magnifications of the boxed regions in C′ and D′, respectively. (E,F) Lineage tracing was performed using *Prox1-CreERT2*;*R26^+/tdTomato^* in *Gata2^+/f^* (E) or *Gata2^f/f^* (F) backgrounds. Tamoxifen was injected at E10.5 to label the PROX1^+^ LVV-ECs and LECs with tdTomato. Subsequently, the embryos were analyzed at E15.5. Whereas LVV-ECs were labeled in control embryos (E, arrows), LVVs were absent and very few labeled cells were observed in the veins of mutant embryos (F). IJV, internal jugular vein; LS, lymph sac; SCV, subclavian vein; SVC, superior vena cava; TM, tamoxifen. (A,B) *n*=6 embryos per genotype; (C,D) *n*=3 embryos and 6 LVV complexes per genotype; (E,F) *n*=3 embryos per genotype. Scale bars: 100 μm (A,B,E,F); 50 μm (C,D); 200 μm (C′); 500 μm (D′); 30 μm (C″,D″).

By E12.5, control embryos displayed LVV-ECs in the venous walls and two-well formed LVVs (Fig. 3A, arrows). In contrast, E12.5 *Gata2^LECKO^* embryos had very few LVV-ECs in the venous walls and lacked clearly defined LVVs (Fig. 3B, arrow). In addition, E12.5 Tg(Prox1-tdTomato) control embryos exhibited two tightly aggregated clusters of tdTomato^high^ LVV-ECs (Fig. 3C, arrows), and SEM of one these clusters (Fig. 3C′) revealed elongated LVV-ECs that formed an opening in the middle (LVV) to permit lymph return to the blood circulation (Fig. 3C″, arrowhead). In contrast, Tg(Prox1-tdTomato);*Gata2^LECKO^* embryos lacked tdTomato^high^ LVV-ECs (Fig. 3D), and SEM of the LVV-forming region (Fig. 3D′) revealed a smooth luminal surface, devoid of LVVs (Fig. 3D″).

Fluorescent reporter proteins such as tdTomato have a long half-life and can remain within cells for several days after the reporter gene is shut off (Muzumdar et al., 2007). However, tdTomato^high^ LVV-ECs in Tg(Prox1-tdTomato);*Gata2^LECKO^* embryos disappear within 12 h (between E12.0 and E12.5), suggesting that LVV-ECs were eliminated either by cell death or by detachment and removal via the bloodstream. To verify the loss of LVV-ECs, we performed lineage tracing using *Prox1-CreERT2*, which is expressed in the lymphatic vasculature, liver and the lens, but not in blood endothelial cells or blood cells (Srinivasan et al., 2007). We generated *Prox1-CreERT2;R26^+/tdTomato^* embryos in a control (wild-type) and *Gata2^f/f^* background, treated pregnant dams with tamoxifen at E10.5, and evaluated embryos at E15.5. *R26^+/tdTomato^* allowed us to lineage trace the PROX1^+^ cells (LECs and LVV-ECs). Whereas entire LVVs were tdTomato^+^ in control embryos (Fig. 3E, arrows), LVVs were absent in *Prox1-CreERT2;Gata2^f/f^;R26^+/tdTomato^* embryos (Fig. 3F). Importantly, the LVV-forming area of *Prox1-CreERT2;Gata2^f/f^;R26^+/tdTomato^* embryos had very few labeled cells (Fig. 3F), consistent with a loss of LVV-ECs in embryos lacking *Gata2*.

LVV-ECs in E12.0 or E12.5 control embryos did not express the proliferation marker phospho-histone 3 (PHH3) (data not shown), indicating that these cells do not proliferate. Therefore, the lack of LVV-ECs in *Gata2^LECKO^* embryos does not reflect impaired proliferation. We observed a few activated Casp3^+^ apoptotic cells within the lymph sacs of *Gata2^LECKO^* embryos, but LVV-ECs did not appear to be labeled by this marker for apoptosis (data not shown). Together, these results indicate that GATA2 is not required for the differentiation of LVV-ECs or for their survival or proliferation. However, we cannot exclude the possibility of cell death with the simultaneous detachment of LVV-ECs into the bloodstream.

In summary, GATA2 regulates the morphology of LVV-ECs, maintains their presence in the valve-forming region, and regulates their morphogenesis into LVVs.

### GATA2 is not necessary for the upregulation of PROX1 and FOXC2 in venous valves, LVs or aortic valves

**Fig. 4.**
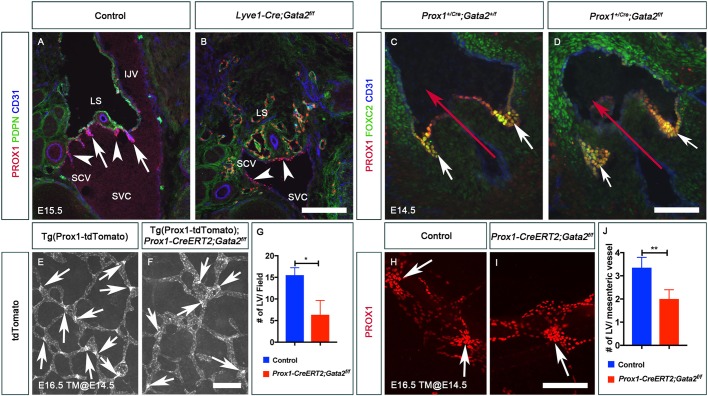
**GATA2 is not required for the differentiation of venous valve-, cardiac valve- and lymphatic valve-forming endothelial cells.** (A) LVVs (arrows) and venous valves (VVs, arrowheads) were seen at the junction of internal jugular vein, subclavian vein and superior vena cava of E15.5 control embryos. (B) LVVs were absent in E15.5 *Lyve1-Cre;Gata2^f/f^* embryos. Although VV-ECs were present in the mutants (arrowheads), they did not invaginate into the veins and did not have the proper morphology of VVs. (C,D) PROX1^+^ FOXC2^+^ endothelial cells (white arrows) were observed on the downstream side of cardiac valves in both control (C) and *Prox1^+/Cre^;Gata2^f/f^* (D) embryos, which lack GATA2 in all PROX1-expressing cells. The red arrow indicates the direction of blood flow. (E-J) Lymphatic vessels in the dorsal skin (E-G) and mesentery (H-J) of E16.5 control and *Prox1-CreERT2;Gata2^f/f^* embryos, which were exposed to tamoxifen at E14.5, were analyzed. Lymphatic valve-forming endothelial cells (LV-ECs) were seen in both control and mutant embryos (arrows). However, LV clusters were reduced in mutants (G,J). IJV, internal jugular vein; LS, lymph sac; SCV, subclavian vein; SVC, superior vena cava; TM, tamoxifen. (A,B) *n*=3 embryos and 6 LVV complexes per genotype; (C,D) *n*=3 embryos per genotype; (E,F,H,I) *n*=3 embryos per genotype. **P<0.01; *P<0.05. Scale bars: 200 μm (A,B,H,I); 100 μm (D); 250 μm (E,F).

GATA2 is also expressed in LVs, venous valves (VVs) and aortic valves (AoVs) (Kazenwadel et al., 2015 and data not shown). Hence, we tested whether GATA2 is necessary for the differentiation of those valvular endothelial cells. VVs of the jugular vein exist close to LVVs at E16.5. VV-forming endothelial cells (VV-ECs) differentiate in this region at around E14.5 (Geng et al., 2016). Control E15.5 embryos displayed LVVs (Fig. 4A, arrows) and developing VVs invaginating into the veins (Fig. 4A, arrowheads). In contrast, *Gata2^LECKO^* E15.5 embryos lacked LVVs, and PROX1^high^ VV-ECs were not invaginating into the veins (Fig. 4B, arrowheads). Expression of FOXC2 was also unaffected in the VV-ECs of *Gata2^LECKO^* embryos (data not shown). Thus, GATA2 is not necessary for the differentiation of VV-ECs.

We deleted *Gata2* using *Prox1^+/Cre^* to remove GATA2 from the PROX1^+^ aortic valve endothelial cells (AoV-ECs) (Srinivasan et al., 2010). PROX1 and *Prox1^+/Cre^* are expressed in AoV-ECs as early as E12.5 (data not shown). As shown in Fig. 4C,D, expression of PROX1 and FOXC2 was unaffected in the AoV-ECs of E14.5 *Prox1^+/Cre^;Gata2^f/f^* embryos. We also did not observe any obvious differences in the expression of PROX1 and FOXC2 in E16.5 *Prox1^+/Cre^;Gata2^f/f^* embryos (data not shown). Thus, GATA2 is not required to upregulate PROX1 and FOXC2 in AoV-ECs or to maintain these cells.

We noticed that, in contrast to *Gata2^LECKO^* embryos, *Prox1-CreERT2;Gata2^f/f^* embryos do not develop blood-filled lymphatic vessels even though they lack LVVs (Fig. 3F). Blood-filled lymphatic vessels could affect LV development (Sweet et al., 2015). Therefore, we analyzed *Prox1-CreERT2;Gata2^f/f^* embryos to investigate the role of GATA2 in LV development. We exposed pregnant dams carrying *Prox1-CreERT2;Gata2^f/f^* embryos to tamoxifen at E14.5 and harvested the embryos at E16.5. Analysis of the skin and mesenteric lymphatic vessels revealed that LV-EC clusters were present in both control and mutant embryos, although those numbers were reduced in the mutants (Fig. 4E-J). In contrast, E18.5 *Prox1-CreERT2;Gata2^f/f^* embryos that were exposed to tamoxifen at E14.5 completely lacked LVs (data not shown). Hence, these results suggest that GATA2 is not necessary for the differentiation of LV-ECs, but it is necessary to maintain those cells.

Together, these results indicate that GATA2 is not necessary for the differentiation of LVV-ECs, VV-ECs, LV-ECs or AoV-ECs or for the upregulation of PROX1 and FOXC2 in those cells. However, GATA2 is necessary to maintain vascular valve endothelial cells (LVV-ECs and VV-ECs) and promote their morphogenesis.

### *EGFL7* and *ANGPT2* are regulated by GATA2 in HLECs

**Fig. 5.**
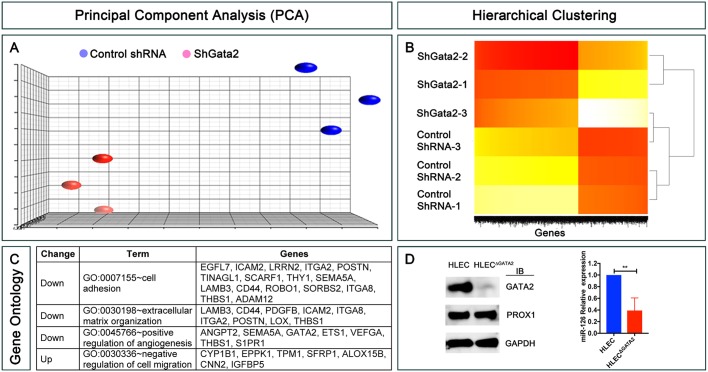
**RNA-seq identifies the targets of GATA2 in primary human LECs.** (A) Principal component analysis (PCA) was performed on RNA-seq data from control shRNA- and shGATA2-infected primary HLECs. A high level of similarity was observed within the groups as indicated by their proximity to each other. (B) Hierarchical clustering shows that approximately 1000 genes were consistently downregulated and 600 genes were upregulated in shGATA2-treated HLECs. (C) GO revealed a list of genes that are likely relevant to the phenotypes observed in mice lacking GATA2. (D) GATA2 was knocked out from a second HLEC line using CRISPR/Cas9. Western blot revealed the lack of GATA2 in the knockout cells (HLEC^ΔGATA2^). In contrast, no obvious differences were observed in the expression of PROX1. Additionally, qRT-PCR revealed the downregulation of miR-126. (A) *n*=3 independent experiments per shRNA; (D) *n*=3 independent experiments (antibiotic selection, western blot and qRT-PCR). **P<0.01.

To identify potential genes regulated by GATA2 *in vivo*, we examined GATA2-dependent gene expression in HLECs. We treated HLECs with lentiviral particles expressing shGFP or shGATA2, harvested them 72 h later and performed RNA-seq. We performed this experiment in triplicate, and principal component analysis confirmed the consistency in gene expression changes among the triplicates (Fig. 5A). We pursued genes for which expression was significantly (*P*<0.05) different between shGFP- and shGATA2-treated HLECs, with log_2_ fold change (FC)>0.5 or log_2_ FC<−0.5. According to these criteria, 1009 genes were significantly downregulated and 617 genes were significantly upregulated upon depletion of *GATA2* in HLECs (Fig. 5B, Table S1). *GATA2* was dramatically downregulated (log_2_ FC=−2.08) in shGATA2-treated HLECs, as expected (Table S1). We did not observe significant changes in the expression of *PROX1*, *FOXC2* or *FLT4*. However, a number of other genes that regulate vascular development were differentially expressed in shGATA2-treated HLECs (Fig. 5C).

Among the top 50 most downregulated genes only *GATA2*, angiopoietin 2 (*ANGPT2*) and EGF-like domain-containing protein 7 (*EGFL7*) are reported to be necessary for embryonic survival and vascular development (Dellinger et al., 2008; Gale et al., 2002; Kuhnert et al., 2008; Wang et al., 2008). EGFL7 (NM_201446) is a secreted protein that was first reported as a regulator of vascular lumen formation (Parker et al., 2004). EGFL7 also regulates blood endothelial cell migration, contractility and adhesion (Charpentier et al., 2013). *EGFL7* is also the host gene for miR-126, which was the first endothelial cell-specific microRNA to be reported (Lagos-Quintana et al., 2002). Interestingly, EGFL7 is not required for the survival of mice as long as miR-126 is intact (Kuhnert et al., 2008). In contrast, most *miR-126^−/−^* embryos die *in utero* with severe edema (Kuhnert et al., 2008; Wang et al., 2008). Importantly, GATA2 was recently reported to regulate EGFL7 and miR-126 in blood vascular endothelial cells (Hartmann et al., 2016). However, the lymphatic vasculature-specific roles of miR-126 remain unknown.

ANGPT2 is a secreted molecule and a ligand of TIE2 (also known as TEK). In blood endothelial cells, ANGPT2 is an antagonist of TIE2. The TIE2/ANGPT2 interaction in blood endothelial cells results in VE-PTP (PTPRB)-mediated downregulation of VE-cadherin (cadherin 5) (Souma et al., 2018). In contrast, in LECs ANGPT2 is an agonist of TIE2 due to the absence of VE-PTP. Deletion of *Angpt2* results in a strain-specific postnatal lethality in mice due to severe chylothorax (Dellinger et al., 2008; Gale et al., 2002). *Angpt2^−/−^* mice lack LVs and have defective cell junctions. Whether ANGPT2 is necessary for LVV development is unknown.

To validate whether miR-126 is a GATA2 target, we used CRISPR/Cas9 to knock out *GATA2* in a distinct HLEC cell line (HLEC-2). Western blotting and DNA sequencing confirmed the deletion of *GATA2* in HLEC^ΔGATA2^ (Fig. 5D, Fig. S4). After extracting miRNAs from the cells we determined by qRT-PCR that miR-126 is significantly downregulated in HLEC^ΔGATA2^ cells compared with controls (Fig. 5D).

In summary, our results indicate that *EGFL7*, miR-126 and *ANGPT2* are potential targets of GATA2 in HLECs.

### *miR-126* is a physiologically important target of GATA2 in the lymphatic vasculature

**Fig. 6.**
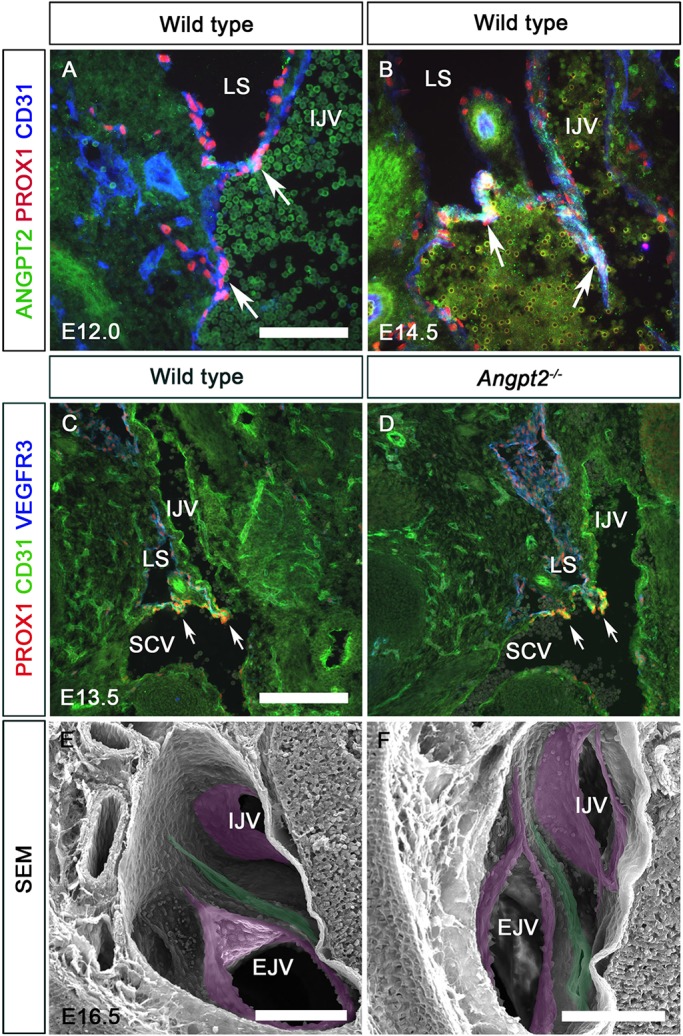
**Angiopoietin2 does not regulate LVV development.** (A,B) ANGPT2 was not expressed in E12.0 LVV-ECs (A, arrows). However, it was expressed in LVV-ECs at E14.5 (B, arrows). (C,D) LVVs developed normally in E13.5 *Angpt2^−/−^* embryos (arrows). (E,F) LVVs (green) and VVs (magenta) developed normally in E16.5 *Angpt2^−/−^* embryos. Lymphovenous valves are pseudocolored in green and venous valves are pseudocolored in pink. IJV, internal jugular vein; EJV, external jugular vein; LS, lymph sac; SCV, subclavian vein. (A-F) *n*=3 embryos and 6 LVV complexes per genotype. Scale bars: 100 μm (A,B,E,F); 200 μm (C,D).

To investigate the physiological relevance of these candidate GATA2 target genes, we compared their expression in the LVV-ECs of E12.0 control and *Gata2^LECKO^* embryos. ANGPT2 was not expressed in LVV-ECs at E12.0 although it appears at E14.5 (Fig. 6). *Angpt2^−/−^* embryos lacked LVs and had defective lymphatic vessel patterning as reported previously (data not shown) (Dellinger et al., 2008; Gale et al., 2002). However, *Angpt2^−/−^* embryos retained normal looking LVVs and VVs (Fig. 6). These observations suggested that GATA2-dependent regulation of *Angpt2* is not involved in LVV and VV development. Hence, we focused our attention on miR-126 for the rest of this work.

**Fig. 7.**
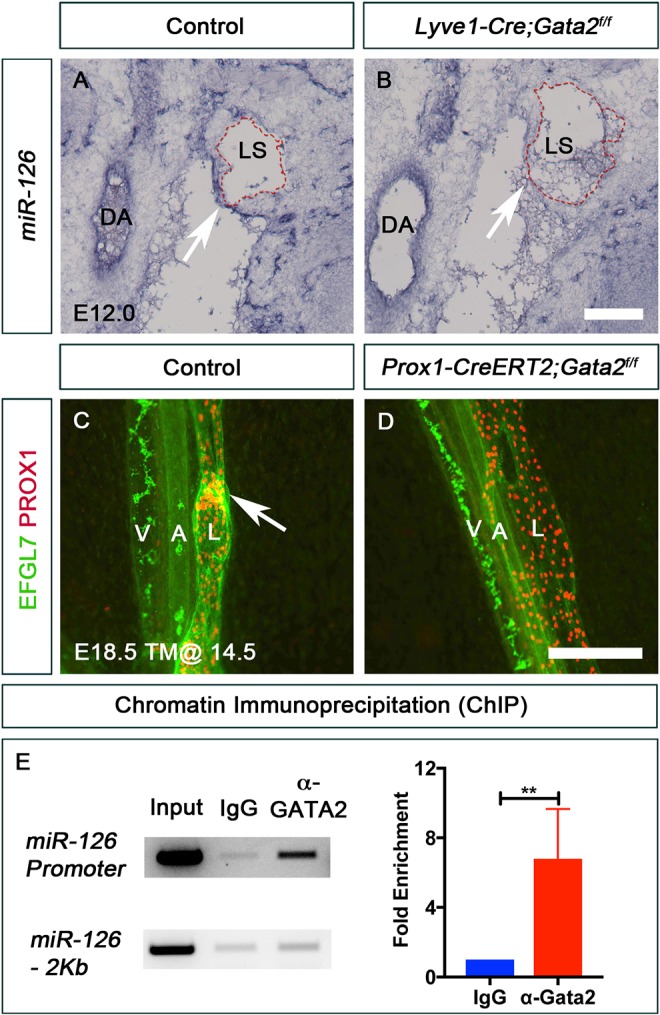
***EGFL7/miR-126* is a target of GATA2.** (A) miR-126 was expressed in the dorsal aorta and in the LVV-ECs (arrow) of control embryos. (B) Expression of miR-126 was downregulated in the LVV-ECs (arrow) of E12.0 *Lyve1-Cre;Gata2^f/f^* embryos. However, no obvious difference in miR-126 expression was observed in the dorsal aorta of mutants. Red dashed line indicates the endothelial layer of the lymph sac. (C) EGFL7 was expressed in the mesenteric arteries, veins and lymphatic vessels of E18.5 control embryos. The strongest expression of EGFL7 was observed in LVs (arrow). (D) Expression of EGFL7 was dramatically downregulated in the mesenteric lymphatic vessels of mice lacking GATA2 in LECs. Also, notice the absence of LVs in the mutant. (E) ChIP revealed that GATA2 strongly associates with the promoter element of the *EGFL7/miR-126* locus. The top gel shows PCR performed using primers flanking the GATA2-binding site. The lower gel shows PCR performed using primers for a non-specific site. The graph compares qPCR signals generated by primers flanking the GATA2-binding site. A, artery; DA, dorsal aorta; L, lymphatic vessel; LS, lymph sac; V, vein. (A,B) *n*=3 embryos and 6 LVV complexes per genotype; (C,D) *n*=3 embryos per genotype; (E) *n*=4. **P<0.01. Scale bars: 250 μm (A,B); 200 μm (C,D).

*In situ* hybridization revealed high expression of *miR-126* in LVV-ECs of E12.0 control embryos but not *Gata2^LECKO^* embryos (Fig. 7A,B, arrows). Similarly, E18.5 control embryos expressed EGFL7 in the LECs of mesenteric lymphatic vessels and in LVs (Fig. 7C, arrow), whereas EGFL7 expression was dramatically reduced in the LECs of E18.5 *Prox1-CreERT2*;*Gata2^f/f^* embryos in which *Gata2* deletion was induced by tamoxifen injection at E14.5 (Fig. 7D). As mentioned previously, the mutants lacked LVs. Thus, GATA2 is required for *EGFL7/miR-126* expression in the developing lymphatic vasculature.

A putative GATA2-binding site (GATAA) is present in the promoter of *EGFL7/miR-126.* GATA2 associates with this regulatory element in primary human umbilical vein endothelial cells (HUVECs) (Hartmann et al., 2016). We performed chromatin immunoprecipitation (ChIP) using an anti-GATA2 antibody and determined that GATA2 associates with this promoter region in HLECs as well (Fig. 7E). These results suggest that *EGFL7/miR-126* is a direct target of GATA2 in the lymphatic vasculature.

**Fig. 8.**
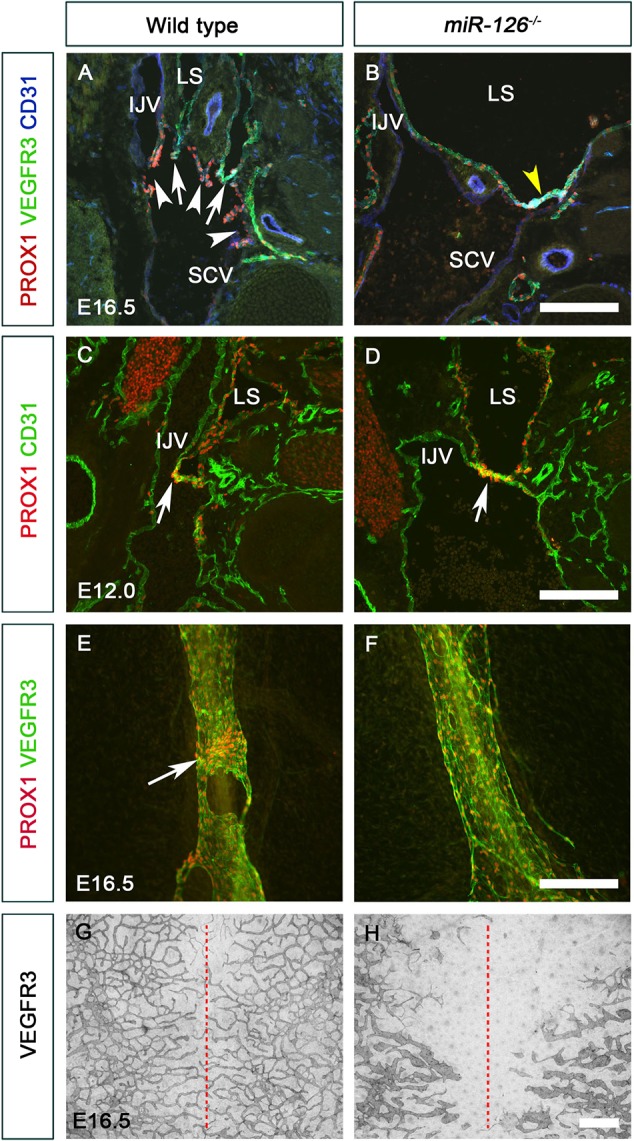
***miR-126^−/−^* embryos are phenotypically similar to mice lacking *Gata2* in LECs.** (A,B) LVVs (arrows) and VVs (white arrowheads) were seen at the junction of the internal jugular vein and the subclavian vein of E16.5 control (A) but not *miR-126^−/−^* (B) embryos. A few PROX1^+^ cells were nevertheless seen at the interface of vein and lymph sacs (B, yellow arrowhead). (C,D) LVV-ECs were observed in both E12.0 control and *miR-126^−/−^* littermates (arrows) indicating that miR-126 is not necessary for the differentiation of LVV-ECs. (E,F) LVs were observed in the mesenteric lymphatic vessels of E16.5 control embryos (E, arrow). PROX1 expression was higher in the LVs compared with LECs. (F) LVs were absent and PROX1 expression was homogeneous in the LECs of *miR-126^−/−^* littermates. (G,H) Lymphatic vessels in the dorsal skin had migrated from the lateral edges to the midline (red dashed lines) in control (G) but not *miR-126^−/−^* (H) embryos. In addition, the lymphatic vessels of the mutants were dilated with fewer branch points. IJV, internal jugular vein; LS, lymph sac; SCV, subclavian vein. (A-D) *n*=3 embryos and 6 LVV complexes per genotype; (E-H) *n*=3 embryos per genotype. Scale bars: 200 μm (A-F); 1000 μm (G,H).

As mentioned previously *Egfl7^−/−^* mice that retain miR-126 are phenotypically normal (Kuhnert et al., 2008). Therefore, we analyzed *miR-126^−/−^* embryos, which display severe edema (Wang et al., 2008). LVVs and VVs were absent in the jugulo-subclavian vein junction of E16.5 *miR-126^−/−^* embryos compared with wild type (Fig. 8A,B). LVV-ECs were present in E12.0 *miR-126^−/−^* embryos (Fig. 8C,D), indicating that *miR-126* is not necessary for the differentiation of LVV-ECs, but is required for their maintenance. *miR-126^−/−^* embryos had dilated mesenteric lymphatic vessels that lacked LVs (Fig. 8E,F), and the lymphatic vessels in the dorsal skin were hypoplastic (Fig. 8G,H).

Overall, these observations demonstrate that GATA2 regulates miR-126 both *in vitro* and *in vivo*. Furthermore, a significant level of phenotypic similarity between *Gata2^LECKO^* and *miR-126^−/−^* embryos, including defects in the maintenance of LVVs and lymphatic vessel patterning indicate that miR-126 is likely a physiologically relevant target of GATA2 in the lymphatic vasculature.

### GATA2 regulates lymphatic endothelial cell junctions via *miR-126*

**Fig. 9.**
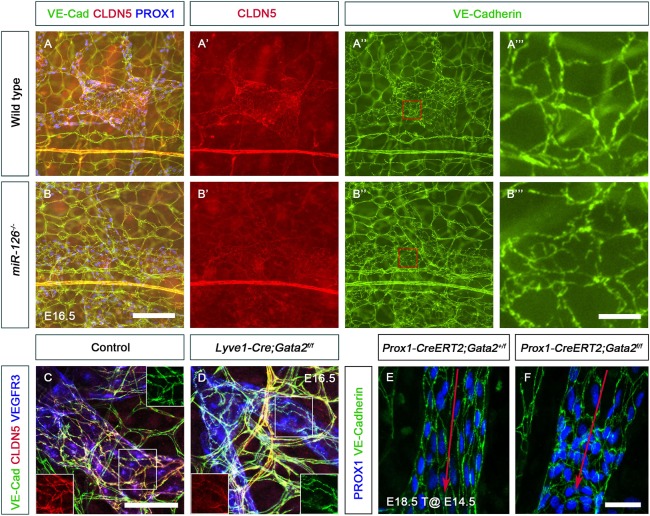
**GATA2 and miR-126 are necessary for the expression of cell junction molecules claudin 5 and VE-cadherin.** (A-B‴) Expression of the tight junction molecule claudin 5 (CLDN5) was dramatically downregulated in the dorsal skin lymphatic vessels of E16.5 *miR-126^−/−^* embryos (B′). The intensity of VE-cadherin staining appeared to be not different between control (A″) and mutant (B″) embryos. However, closer inspection revealed that VE-cadherin was uniformly expressed along the cell boundaries of control embryos (A‴), but was localized in a ‘zig-zag’ pattern in mutant (B‴) embryos. A‴ and B‴ are magnifications of the boxed regions in A″ and B″, respectively. (C,D) The red and green channels from the central boxed region are shown as insets. Claudin 5 was downregulated (D, left inset) and VE-cadherin had a defective localization with gaps (D, right inset) in the dorsal skin lymphatic vessels of E16.5 *Lyve1-Cre;Gata2^f/f^* embryos. (E) The LECs of mesenteric lymphatic vessels were elongated in the direction of lymph flow (red arrow) in E18.5 control embryos. VE-cadherin was uniformly expressed around the cell boundaries of control LECs. (F) In contrast, the LECs were misaligned and VE-cadherin appeared to be mislocalized in embryos lacking GATA2. *n*=3 embryos per genotype. Scale bars: 200 μm (A,B); 25 μm (A‴,B‴); 50 μm (C-F).

To identify the relevant targets of miR-126, we performed RNA-seq in HLECs expressing an ‘miR-126 sponge’ (Gentner et al., 2009; Lechman et al., 2012) to sequester *miR-126* from its endogenous targets (Table S1). Using the same criteria described above, we identified 1058 genes that were upregulated and 873 genes that were downregulated by the presence of the miR-126 sponge. *SPRED1* and *PIK3R2*, which are reported targets of miR-126 in blood endothelial cells, were not in the list (Fish et al., 2008; Kuhnert et al., 2008; Wang et al., 2008). *PROX1*, *FOXC2*, *FLT4* and *GATA2* were also not found in this list. By comparing these genes with the GATA2-regulated genes we identified 125 shared downregulated genes and 72 shared upregulated genes (Fig. S5). DAVID gene annotation was used to classify the shared genes (Huang et al., 2009a,b). Sixteen clusters were observed among the downregulated genes with 42 membrane-associated proteins constituting the largest group. Nine clusters were observed among upregulated genes, which included the keywords Membrane, Cytoskeleton, Microtubule, Metalloprotease, Rap1 signaling and Cell junctions. All of the terms identified among upregulated and downregulated genes are relevant to the regulation of vascular integrity (Chrzanowska-Wodnicka, 2017; Dudek and Garcia, 2001). Therefore, we analyzed the expression of cell junction molecules in *Gata2^LECKO^* and *miR-126^−/−^* embryos. Claudin 5 expression was dramatically downregulated in the lymphatic vessels of E16.5 *miR-126^−/−^* embryos (Fig. 9A,A′,B,B′). Additionally, whereas VE-cadherin was uniformly expressed along the cell junctions of control embryos, it displayed discontinuous expression in *miR-126^−/−^* embryos (Fig. 9A″,A‴,B″,B‴). We identified identical defects in VE-cadherin and claudin 5 expression in E16.5 *Gata2^LECKO^* embryos (Fig. 9C,D). In addition, VE-cadherin expression was disorganized in the mesenteric lymphatic vessels of E18.5 *Prox1-CreERT2;Gata2^f/f^* embryos that were exposed to tamoxifen at E14.5 (Fig. 9E,F).

Thin sections of LVV-ECs from E12.0 control and *Gata2^LECKO^* embryos did not reveal any obvious differences in VE-cadherin or claudin 5 expression (Fig. S1M-P). However, LVV-ECs delaminate from the walls of veins at E12.0 before reassembling in multiple layers to form mature LVVs at E12.5 (Geng et al., 2016). Such a rapid morphogenesis of LVV-ECs is likely to involve dramatic reorganization of cell junctions. Furthermore, the deletion of VE-cadherin from the lymphatic vasculature was recently reported to inhibit the formation or maintenance of LVVs and LVs (Hagerling et al., 2018; Yang et al., 2019). Hence, we are tempted to speculate that a defect in the reorganization of cell junctions might be the cause of LVV-EC disappearance in E12.5 *Gata2^LECKO^* embryos.

**Fig. 10.**
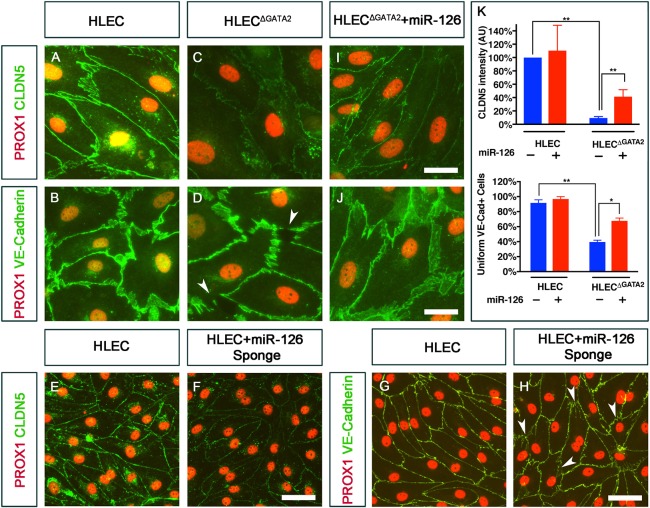
**GATA2 regulates the expression of claudin 5 and VE-cadherin in HLECs in a miR-126-dependent manner.** (A,B) Claudin 5 (A) and VE-cadherin (B) were uniformly expressed in the cell boundaries of control HLECs. (C,D) In contrast, claudin 5 was dramatically downregulated in HLEC^ΔGATA2^ (C) and VE-cadherin was expressed in a zig zag pattern (arrowheads) on the cell boundaries of HLEC^ΔGATA2^ (D). (E-H) Expression of a miR-126 sponge using lentivirus strikingly downregulated claudin 5 (F) and caused disruptions in VE-cadherin localization (H, arrowheads) in HLECs. (I,J) Overexpression of miR-126 using lentiviral particles significantly rescued the expression of claudin 5 (I) and VE-cadherin (J) expressions in HLEC^ΔGATA2^. (K) Quantification of the intensity of claudin 5 expression and the number of cells with uniform expression of VE-cadherin. (A-D,E-H,I) *n*=3; (J) *n*=2. **P<0.01; *P<0.05. Scale bars: 25 μm (A-F); 50 μm (G-J).

Given that the lymphatic vessels of E16.5 *Gata2^LECKO^* and *miR-126^−/−^* embryos had defective cell junctions, we examined claudin 5 and VE-cadherin expression in HLECs. Claudin 5 and VE-cadherin were uniformly expressed around the entire periphery in ∼80% of control HLECs (Fig. 10A,B). In contrast, the intensity of claudin 5 expression was dramatically reduced in HLEC^ΔGATA2^ cells (Fig. 10C). In addition, the localization of VE-cadherin was defective in HLEC^ΔGATA2^ cells with numerous gaps (Fig. 10D, arrowheads). To determine whether miR-126 also influences claudin 5 expression in HLECs, we overexpressed the ‘miR-126 sponge’ (Lechman et al., 2012) in HLECs, and observed a significant reduction in claudin 5 expression and defective VE-cadherin localization (Fig. 10E-H). Thus, both GATA2 and *miR-126* are regulators of claudin 5 expression and VE-cadherin localization *in vitro* and *in vivo*.

To determine whether GATA2 regulates claudin 5 via miR-126, we overexpressed *miR-126* in HLEC^ΔGATA2^ cells using lentiviral particles (Amendola et al., 2009). We observed a partial, yet significant rescue of claudin 5 expression in HLEC^ΔGATA2^ expressing *miR-126*. In addition, miR-126 significantly rescued the localization of VE-cadherin at the cell junctions (Fig. 10I-K). These results suggest that GATA2 regulates adherens and tight junctions in LECs through miR-126.

## DISCUSSION

**Fig. 11.**
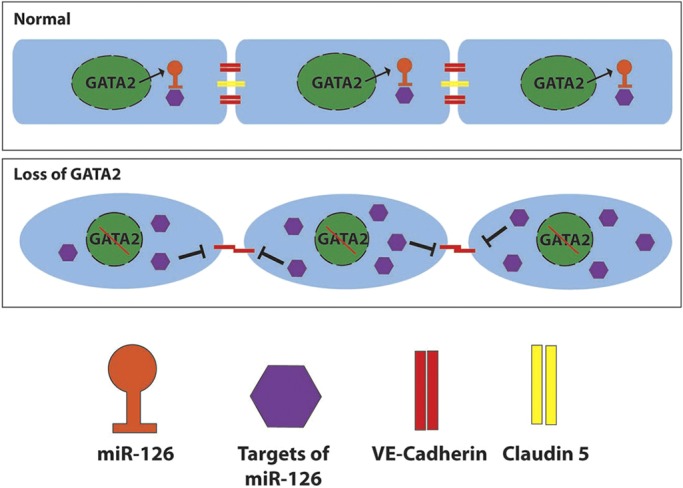
**GATA2 regulates endothelial cell junctions through miR-126.** Under normal conditions, GATA2 enhances the expression of miR-126 in the lymphatic vasculature. In the absence of GATA2, the targets of miR-126 are upregulated, which results in the downregulation of claudin 5 and mislocalization of VE-cadherin. Consequently, lymphatic vascular morphogenesis is defective due to the abnormal shape of LECs and LVV-ECs.

In this work, we have discovered that although GATA2 is not necessary for LVV-EC differentiation, it is required for their maintenance. Furthermore, GATA2 is important for LVV-ECs and LECs to align appropriately with respect to the direction of fluid flow. GATA2 activates the expression of *miR-126* in LVV-ECs and LECs. The lymphatic vascular defects of mice lacking GATA2 or *miR-126* are strikingly similar, and both GATA2 and *miR-126* are necessary for the expression of the cell junction molecules claudin 5 and VE-cadherin. Importantly, *miR-126* could significantly rescue cell junction defects in HLECs lacking GATA2. Based on our results, we propose a model in which GATA2 regulates LVV morphogenesis and lymphatic vascular maturation by maintaining proper cell junctions via *miR-126* (Fig. 11).

Valves normally develop at locations of disturbed flow. Hence, pioneering work by Sabine et al. proposed OSS as the most upstream regulator of valve development (Sabine et al., 2012). Significant advances have been made since this initial report. We showed that Wnt/β-catenin signaling enhances the expression of FOXC2 and GATA2 in response to OSS (Cha et al., 2016), and that PROX1 is necessary for the activity of Wnt/β-catenin signaling (Cha et al., 2018). Elegant studies have shown that GATA2 enhances the expression of FOXC2 in an OSS-dependent manner (Kazenwadel et al., 2015; Sweet et al., 2015). Despite this knowledge, the precise role of OSS in valve development is unknown. Cytoskeleton, cell-cell and cell-matrix interactions are crucial regulators of mechanotransduction (Hahn and Schwartz, 2009; Ingber, 2006). Therefore, our finding that GATA2 regulates VE-cadherin and claudin 5 expression through miR-126 provides a mechanistic explanation for GATA2-mediated mechanotransduction. Surprisingly, GATA2 is not necessary for the upregulation of FOXC2 expression in LVV-ECs *in vivo*. Therefore, we suggest that the OSS/GATA2/FOXC2 axis is not necessary for the differentiation of valvular endothelial cells. Instead, OSS might be important for lymphatic vessel patterning and the maintenance of FOXC2 expression in valvular endothelial cells. In line with this model, the mechanosensory ion channel PIEZO1 was recently shown to be necessary for LV development (Choi et al., 2019; Nonomura et al., 2018). Just like GATA2, PIEZO1 is not necessary for the differentiation of LV-ECs (Nonomura et al., 2018). Instead, PIEZO1 maintains LV-ECs and regulates their morphogenesis into LVs. Similar observations were made in mice lacking VE-cadherin (Yang et al., 2019).

Several interesting questions arise from our work for future exploration. Recently, Kontarakis et al. reported that they do not observe any obvious edema or lymphatic defects in *miR-126^−/−^* embryos (Kontarakis et al., 2018). However, we consistently observe severe edema and lymphatic vascular defects in *miR-126^−/−^* embryos (data not shown). The reasons for these phenotypic differences remain to be determined. Neither GATA2 nor *miR-126* regulates claudin 5 at the mRNA level (Table S1; data not shown). Hence, how *miR-126* regulates claudin 5 is currently unknown. The mechanisms behind the abnormal patterning of VE-cadherin are also not known. It will be of interest to generate mice lacking the cell junction molecule claudin 5, to determine whether they recapitulate any of the phenotypes of *Gata2^LECKO^* and *miR-126^−/−^* embryos. Future work should also address whether modulators of cell-ECM interaction and cadherin stability such as ADAM19 and MMP15, which are significantly upregulated in shGATA2- and miR-126 sponge-treated HLECs, play physiologically important roles in the development of the lymphatic vasculature.

How GATA2 regulates blood-lymph separation is not yet clear. LVV-ECs do not form until E12.0 (Geng et al., 2016; Srinivasan and Oliver, 2011). However, the lymph sacs of E11.5 *Gata2^LECKO^* embryos are blood filled (data not shown). Furthermore, as mentioned previously, *Prox1-CreERT2;Gata2^f/f^* embryos did not have blood-filled lymphatics despite the absence of LVVs (Fig. 3E,F). These observations suggest that GATA2 is regulating blood-lymph separation through an LVV-independent mechanism.

Platelet-expressed CLEC2 and LEC-expressed podoplanin play a crucial role in blood-lymph separation (Bertozzi et al., 2010; Fu et al., 2008). Expression of podoplanin is not affected in *Gata2^LECKO^* embryos, and platelets are present in E16.5 *Gata2^LECKO^* embryos (data not shown). Together, these results suggest that GATA2 regulates blood-lymph separation independently of the platelet/LEC interaction. As GATA2 regulates the expression of numerous genes in blood endothelial cells, and as LECs originate predominantly from embryonic veins, we are tempted to speculate that GATA2 might be regulating blood-lymphatic separation by maintaining blood vascular integrity during the migration of LECs from the veins.

Only some patients with mutations in GATA2 will develop lymphedema. A subset of mice lacking one allele of *Gata2* in endothelial cells display a dilated thoracic duct with reduced lymph flow (Kazenwadel et al., 2015), but they have a normal lifespan and do not develop any obvious symptoms of the human disease, such as leukemia, bacterial infections or warts (our unpublished observations). Compared with humans, mice experience less severe gravitational load in the lymphatic vessels of their limbs (Castorena-Gonzalez et al., 2018), which might underlie the absence of obvious lymphatic defects in *Gata2^+/−^* mice. Alternative explanations are also possible. Secondary mutations in *ASXL1* are frequently observed in Emberger syndrome patients who develop MDS/AML (Crispino and Horwitz, 2017). Similarly, a ‘second hit’ in the targets of GATA2, such as miR-126, might be required to trigger the onset of lymphedema in human patients. Non-coding RNAs are powerful biomarkers of human disease owing to their ability to be detected in bodily fluids (Van Roosbroeck et al., 2013). Whether circulating miR-126 levels might predict the onset of lymphedema in Emberger syndrome patients needs to be determined.

Finally, several miRNA mimics and miRNA inhibitors have entered Phase I, Phase II and preclinical trials and approaches to deliver them are rapidly improving (Rupaimoole and Slack, 2017). Hence, we are excited about the possibility that miR-126 might one day be used to treat lymphedema in Emberger syndrome patients.

## MATERIALS AND METHODS

### Cells

We used de-identified primary human lymphatic endothelial cells (HLECs) for experiments. HLEC-1 were from Lonza (CC-2812) and were used for RNA-seq analysis with shGATA2. Drs Young-Kwon Hong and Donwong Choi provided the HLECs that were used for RNA-seq experiments performed using miR-126-sponge (Choi et al., 2019, 2017a,b, 2016). HLEC-2 (Lonza, CC-2516) were used for all other experiments. HLECs were grown on fibronectin-coated plates or glass slides and were maintained in EBM2 media from Lonza. All experiments were conducted using passage 5-6 cells. HLECs were treated as potential biohazards and were handled according to institutional biosafety regulations.

### Mice

*Prox1^+/Cre^* (Srinivasan et al., 2010), *Gata2^f/f^* (Charles et al., 2006), *miR-126^−/−^* (Wang et al., 2008), *Prox1-CreERT2* (Srinivasan et al., 2007), Tg(Prox1-tdTomato) (Gong et al., 2003), *Lyve1-Cre* (Pham et al., 2010), Tie2-Cre (Kisanuki et al., 2001), *R26^+/tdTomato^* (Madisen et al., 2010) mice were described previously*. Prox1^+/Cre^* mice were maintained in NMRI background. Other mice were maintained in C57BL6 or C57BL6/NMRI mixed backgrounds. We used both male and female mice for the experiments. All mice were housed and handled according to the institutional IACUC protocols.

### Chromatin immunoprecipitation

ChIP assays were performed using EZ-ChIP kit (Millipore/Sigma) according to the manufacturer's instructions. Around 1.0×10^7^ HLECs were used per ChIP. Briefly, HLECs were grown on culture dishes at around 100% confluence. Subsequently, HLECs were fixed in 1% formaldehyde for 10 min at room temperature and glycine at a final concentration of 0.125 M was added for 5 min. Cells were washed with 20 ml of ice-cold PBS twice (10 min each) and harvested. Cells were lysed and sonicated as previously described (Cha et al., 2016, 2018).

Chromatin immunoprecipitation was performed using 3.0 μg of goat anti-mouse GATA2 (AF2046, R&D Systems) or 1.0 μg of normal goat IgG antibody (AB-108-C, R&D Systems). Following ChIP, PCR or q-PCR was performed using primers flanking the predicted GATA2-binding site or a control site within EGFL7/miR-126 promoter. The primers for the site around the GATA2-binding site are 5′-CAATCCCGATTACCCAGGACG-3′ and 5′-GGAGATGGACCCTAGCCCTT-3′. The primers for the control site are 5′-TTTGGAAATGGAGGCCTGGAG-3′ and 5′-CACTGGGTCACTGCTGAGTTC-3′. The anti-GATA2 to IgG q-PCR signal ratio at the GATA2-binding site was used to estimate GATA2/DNA interaction.

### Immunohistochemistry of tissues

Immunohistochemistry on sections was performed according to our previously published protocols (Cha et al., 2016, 2018; Geng et al., 2016). Briefly, freshly collected embryos were washed in 1× PBS and fixed in 4% paraformaldehyde (PFA) overnight at 4°C. Subsequently, the embryos were washed three times (10 min each) in cold PBS, incubated in 15% sucrose overnight at 4°C and then in 30% sucrose at 4°C until fully submerged in the solution. Embryos were then cryo-embedded in OCT solution (Sakura). Cryosections (12 μm thick) were prepared using a cryotome (Thermo Fisher Scientific, model: HM525 NX) and immunohistochemistry was performed using the indicated antibodies. E11.5 embryos were sectioned in a transverse orientation and E12.0-E16.5 embryos were sectioned frontally. Several consecutive sections were analyzed to determine the presence or absence of LVVs and VVs.

Whole-mount immunohistochemistry using embryonic skin or guts was performed according to our previous protocol (Cha et al., 2016, 2018). Either whole embryos or isolated guts were washed in 1× PBS and fixed in 1% PFA for 1 h to overnight (depending on the antibody) at 4°C. Subsequently, the dorsal skins were isolated, washed and samples were immunostained using the iDISCO protocol (Renier et al., 2014). Samples were visualized and analyzed as described previously (Cha et al., 2016, 2018).

### Immunostaining of cells

Cells were fixed in 1% PFA at room temperature for 30 min. Cells were subsequently permeabilized with 0.3% Triton X-100 for 10 min at room temperature, then washed with PBST (PBS+0.1% Triton X-100) and blocked in 0.5% BSA PBST for 1 h at room temperature. Samples were incubated with primary antibodies at 4°C overnight. Samples were then washed with PBST and incubated with secondary antibodies for 2 h at room temperature, and then washed with PBST three times (10 min each), mounted and visualized as previously described (Cha et al., 2016, 2018).

### Western blot

Control HLEC or HLEC^ΔGATA2^ were grown in 12-well plates at ∼100% confluency. Cells were harvested with lysis buffer and western blots were performed following a standard protocol (Cha et al., 2016).

### Antibodies

Primary antibodies for immunohistochemistry were: rabbit anti-PROX1 (11-002, Angiobio), goat anti-human PROX1 (AF2727, R&D Systems), sheep anti-mouse FOXC2 (AF6989, R&D Systems), goat anti-mouse VEGRF3 (AF743, R&D Systems), rat anti-mouse CD31 (553370, BD Pharmingen), goat anti-mouse ITGA9 (AF3827, R&D Systems), rat anti-mouse VE-cadherin (550548, BD Pharmingen), hamster anti-mouse PDPN (127401, Biolegend), rat anti-mouse ITGA5 (553319, BD Pharmingen), goat anti-mouse GATA2 (AF2046, R&D Systems), rabbit anti-mouse CX37 (40-4200, Life Technologies), rabbit anti-mouse LAMA5 (Ab11575, Abcam), rabbit anti-human fibronectin (ab2413, Abcam), goat anti-human ANGPT2 (AF623, R&D Systems), goat anti-mouse EGFL7 (AF3089, R&D Systems), rabbit anti-mouse CLDN5 (34-1600, Thermo Fisher Scientific), rabbit anti-mouse LYVE-1 (11-034, Angiobio).

Secondary antibodies for immunohistochemistry were: Cy3-conjugated donkey anti-rabbit, Cy3-conjugated donkey anti-sheep, and Cy5-conjugated donkey anti-rat antibodies (Jackson ImmunoResearch Laboratories), and Alexa 488-conjugated donkey anti-goat, Alexa 488-conjugated goat anti-chicken and Alexa 488-conjugated donkey anti-rat (Life Technologies).

Primary antibodies for western blotting were: mouse anti-β-actin (A5441, Sigma-Aldrich), goat anti-human PROX1 (AF2727, R&D Systems), goat anti-mouse GATA2 (AF2046, R&D Systems) and rabbit anti-human GAPDH (PAB13195, Abnova).

HRP-conjugated secondary antibodies for western blotting were: goat anti-mouse IgG, goat anti-rabbit IgG, donkey anti-goat IgG and donkey anti-sheep IgG (Santa Cruz Biotechnology).

### *In situ* hybridization

We used a kit to detect mmu-miR-126-3p by *in situ* hybridization (339111, Qiagen). Briefly, we fixed the embryos in 4% PFA overnight at 4°C. They were then soaked in sucrose, embedded in OCT and sectioned as described above. The sections were fixed in 4% PFA for 10 min at room temperature and washed in PBS. Subsequent steps were performed according to manufacturer's instructions.

### Scanning electron microscopy

SEM was performed according to our previous protocol (Geng et al., 2016; Geng and Srinivasan, 2018).

### Knockdown of GATA2

shGATA2 (TTAACAGGCCACTGACCATGAAGAAGGAA) was cloned into a pLV plasmid. Cyagen Bioscience (Santa Clara, CA, USA) generated the lentiviral particles using LentiPAC 293 cells. HLECs were seeded at 50-60% confluence on fibronectin-coated plates. The following day, cells were infected with equal amounts of shControl or shGATA2 virus according to the manufacturer's protocol for 4-6 h in Opti-MEM medium and then changed to regular EBM2 media. After 2-3 days cells were harvested with Trizol (Invitrogen) for RNA-seq study.

### Knockout of GATA2 using CRISPR/Cas9

sgRNA1 (GGTCTGGGTGCAGACGGCAA), sgRNA2 (ATGCCAACCCCGCTCACGCG) and Cas9 were cloned into a pLV plasmid with puromycin selection marker. The translational start site ATG of *GATA2* is located between the recognition sites of sgRNA1 and sgRNA2. Cyagen Bioscience (Santa Clara, CA, USA) generated the lentiviral particles using LentiPAC 293 cells. HLEC-2 were seeded at 50-60% confluence on fibronectin-coated plates. The following day, cells were infected with an equal amount of control or GATA2 CRIPSPR/Cas9 recombinant lentiviral particles according to the manufacturer's protocol for 4-6 h in Opti-MEM medium and then changed to regular EBM2 media. After 24 h, cells were treated with 0.5 μg/ml puromycin to select the cells. After 3 days 0.5 μg/ml puromycin treatment, almost all non-infected HLECs were dead. We used 5 days of 0.5 μg/ml puromycin treatment for selecting HLEC^ΔGATA2^.

A gene-specific primer pair was used that could cover both sgRNA1 and sgRNA2 sequences, generating 330-bp-long amplicons. The resulting PCR amplicons were purified using the MinElute PCR purification kit (Qiagen). Sequencing library was constructed from 100 ng DNA and approximately 50-100,000 300-base read pairs were generated on an Illumina MiSeq platform. GeneWiz performed library preparation, sequencing and bioinformatics analysis.

A total of 66,473 reads were aligned to the reference sequence. Sequences that occurred with a frequency of five or more were used for further analysis, and a total 63,526 sequences fit this criterion. Indels were detected in 62,864 sequence reads (∼99%). There were 62,808 sequences with deletions and 42,334 reads (67%) harbored a 231 bp deletion between the two targets.We also detected 232-bp- and 248-bp-long deletions at lower frequencies (13.2% and 1.2%, respectively). There were 2399 sequences (∼3.8%) with two deletions (12 bp and 19 bp) within the sgRNA1 and sgRNA2, respectively.

### miR-126 sponge and miR-126 overexpression

pSFFV plasmids to sequester miR-126 or overexpress miR-126 were reported previously (Amendola et al., 2009; Gentner et al., 2009). Cyagen Bioscience generated the lentiviral particles using LentiPAC 293 cells. HLECs were seeded at 50-60% confluence on fibronectin-coated plates or glass slides. The following day, cells were infected with equal amounts of control, miR-126 sponge or miR-126 overexpression virus for 4-6 h according to the manufacturer's protocol using EBM2 medium and then changed to fresh medium. After 2-3 days, cells were harvested for appropriate study.

### miR-126 isolation and quantitative real-time PCR

MicroRNA along with total RNA was isolated from HLECs using QIAzol lysis reagent (Qiagen) according to the manufacturer's instructions. The cDNA was synthesized from total RNA (0.1-1.0 μg) with the miScript II RT Kit (Qiagen). qRT-PCR was performed using the miScript SYBR Green PCR Kit (Qiagen) in a CFX96 Real-Time System (Bio-Rad). miR-126 expression levels were normalized to *U6.* Pre-designed primers for miR-126 and U6 were purchased from Qiagen (MS00003430 and MS00033740, respectively).

### RNA-seq analysis

Total RNA was purified from HLECs infected with shGATA2- or control shRNA-expressing lentivirus particles. RNA was subjected to ribosomal RNA depletion followed by Truseq stranded total RNA library preparation according to the manufacturer's instructions (Illumina). RNA from miR-126 sponge-treated HLEC-2 were processed using the NEB Ultra II directional RNA Library kit for Illumina. The resulting RNA-seq libraries were analyzed on an Illumina HiSeq sequencing platform.

The obtained sequencing reads were mapped with the bowtie2 algorithm using the RefSeq annotations (hg19 genome build) (Langmead and Salzberg, 2012). We utilized the RNA-seq analysis work flow within the Partek Genomics Suite (Partek Incorporated) for quantification and statistical analysis (ANOVA) of the transcriptome data. We identified those transcripts that exhibited statistically significant differential expression in the shGata2 samples compared with the shControl samples. We rank ordered the two lists based on the expression level and magnitude of change. Using these rank-ordered lists, we performed gene ontology (GO) analysis for enriched biological terms (Eden et al., 2009). The genes commonly regulated by GATA2 and miR-126 were analyzed using the functional annotation platform of DAVID (Huang et al., 2009a,b).

### Statistical analysis

For biochemical analysis, *n* indicates the number of times the experiments were performed and for histological analysis *n* indicates the number of embryos analyzed per genotype. VE-cadherin expression analysis in HLEC^ΔGATA2^ following miR-126 overexpression was performed twice. All other experiments were performed at least three times or more. Data are presented as mean±s.e.m. GraphPad Prism 7 software was used to perform the statistical analysis. Data were analyzed by unpaired, two-tailed Student's *t-*test. *P*<0.05 was considered significant.

## Supplementary Material

Supplementary information

Reviewer comments
