# Supplementary material for: GATA2 controls lymphatic endothelial cell junctional integrity and lymphovenous valve morphogenesis through miR-126
Source: Development. 2019 Nov 5;146(21):dev184218. doi: 10.1242/dev.184218 (PMC6857586; doi:10.1242/dev.184218)
Supplement: Supplementary information [file develop-146-184218-s1.pdf]

Supplementary Figures

Fig. S1

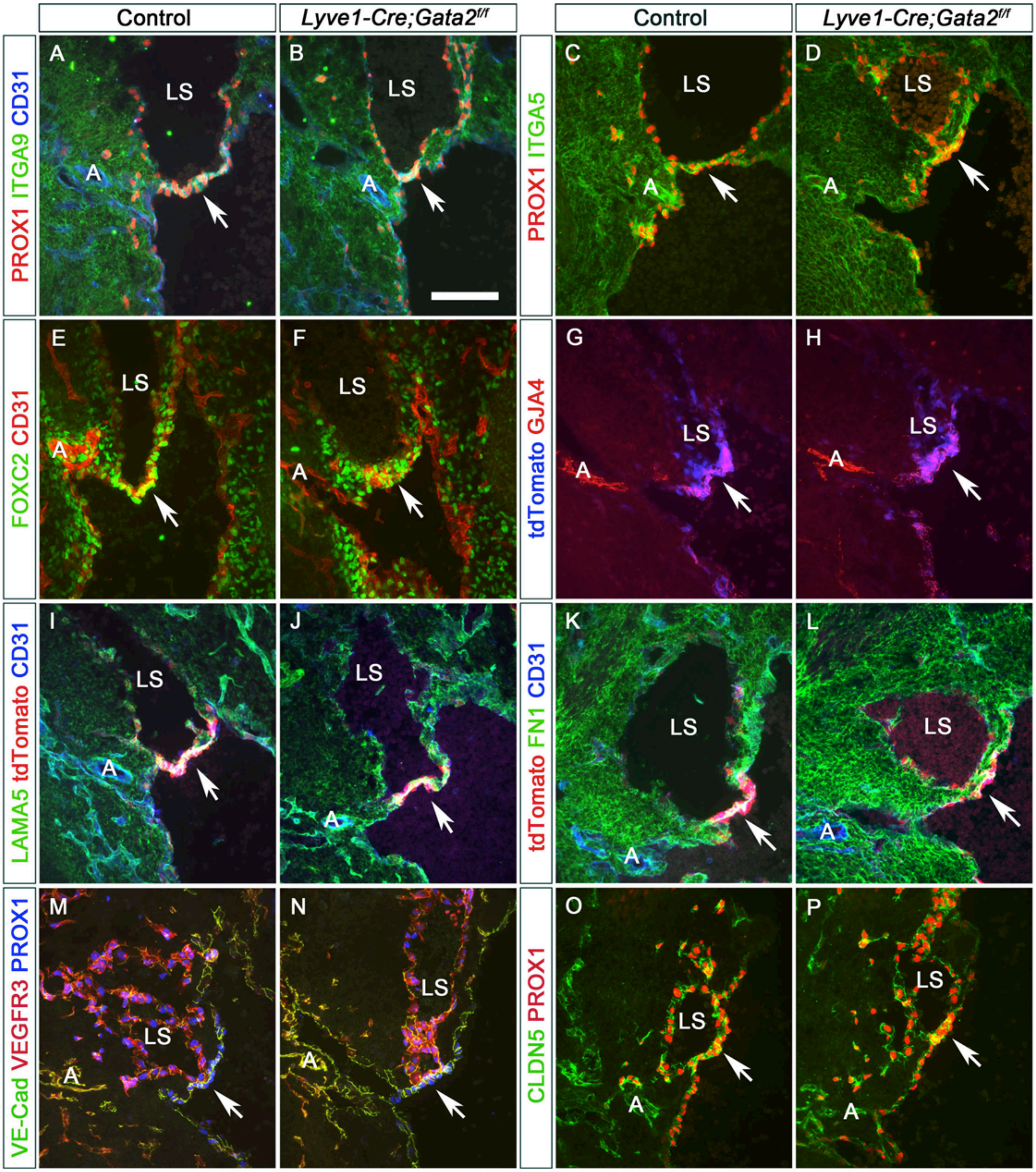

Fig. S1: LVV-ECs, ECM and cell junction molecules are normally expressed in E12.0 embryos lacking GATA2.

E12.0 Wild type and *Lyve1-Cre;Gata2*$^{f/f}$ littermates or Tg(Prox1-tdTomato) and Tg(Prox1-tdTomato); *Lyve1-Cre;Gata2*$^{f/f}$ littermates were frontally sectioned and IHC was performed for LVV-EC markers (PROX1, tdTomato, FOXC2, ITGA5, ITGA9), ECM components (FN1, LAMA5), and cell junction proteins (CD31, VE-Cadherin, GJA4, CLDN5). No obvious differences were observed between control and mutant samples.

Abbreviations: A, artery; LS, lymph sac.

Measuring bar: (A-P) 100 µm.

Statistics: n=3 embryos and 6 LVV complexes per genotype per antibody.

Fig. S2

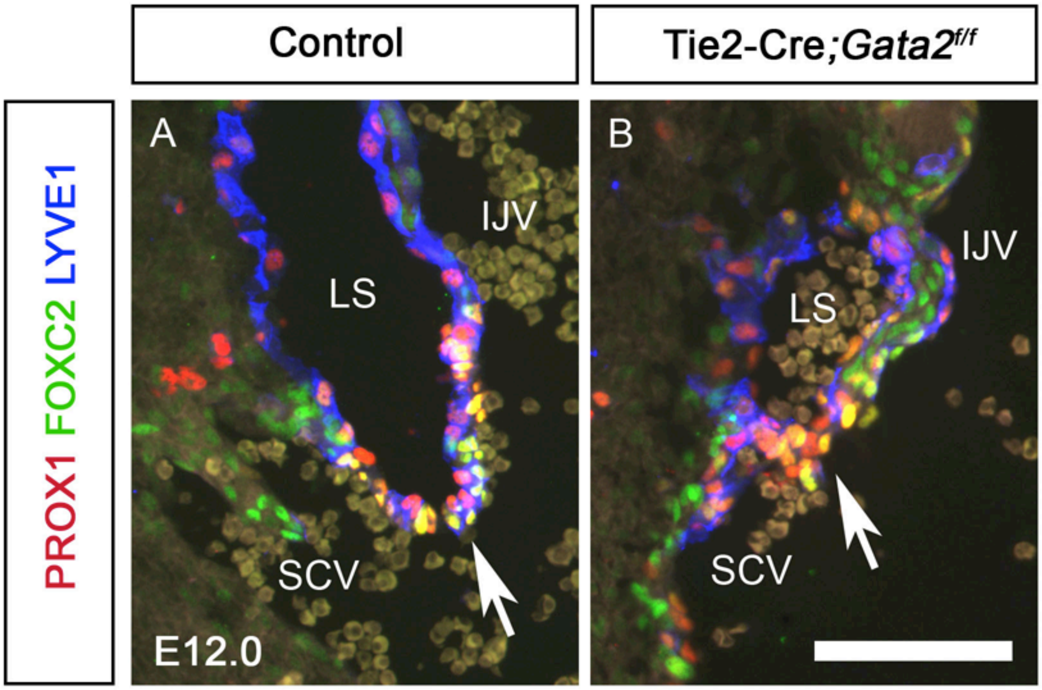

Fig. S2: LVV-ECs are present in E12.0 Tie2-Cre;*Gata2*$^{f/f}$ embryos

PROX1^{+}FOXC2^{+} LVV-ECs are present in both E12.0 control (A, arrow) and mutant embryos lacking GATA2 in all endothelial cells (B, arrow).

Abbreviations: IJV, internal jugular vein; SCV, subclavian vein; LS, lymph sac.

Measuring bar: (A, B) 100 µm.

Statistics: n=3 embryos and 6 LVV complexes per genotype.

Fig. S3

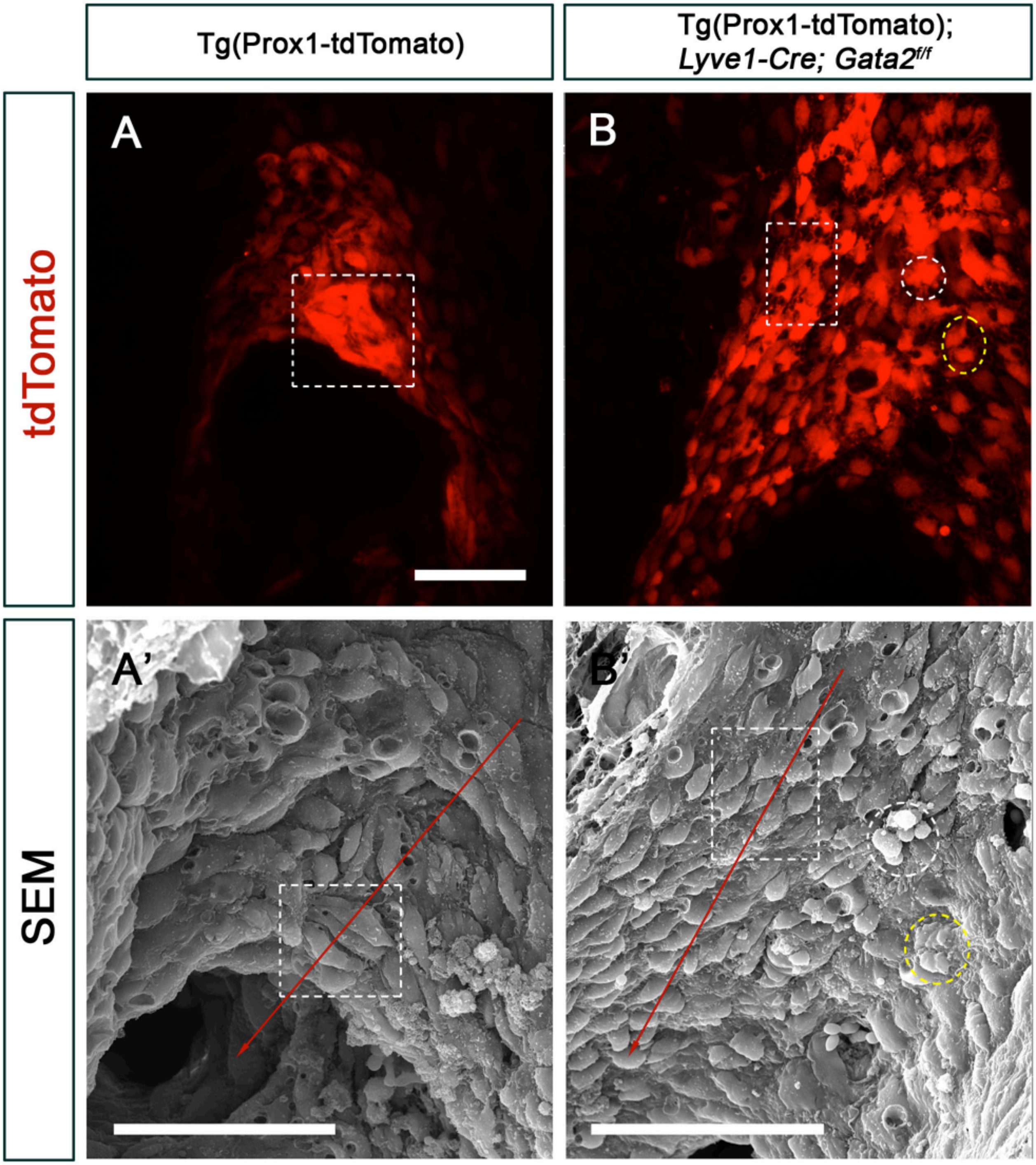

Tg(Prox1-tdTomato)
Tg(Prox1-tdTomato); Lyve1-Cre; Gata2f/f
tdTomato
SEM
A
B
A'
B'

Fig. S3: LVV-ECs of E12.0 *Gata2*^{LECKO} embryos are dysplastic and do not align properly with respect to blood flow

(A, B) E12.0 Tg(Prox1-tdTomato) or Tg(Prox1-tdTomato); *Lyve1-Cre; Gata2*$^{f/f}$ embryos were sagittally sectioned along the internal jugular vein and the tdTomato^{+} LVV-ECs were imaged by confocal microscopy. The same samples were then reprocessed and analyzed by SEM (A', B'). The cells in fluorescent microscopy images that correlate with cells in the SEM images are outlined.

(A') LVV-ECs of control embryos were elongated and compactly aggregated with each other. They were also aligned perpendicular to the direction of blood flow (red arrow). (B') In contrast, LVV-ECs of mutant embryos appear round and dispersed (see cells within the rectangular box) and appeared to be aligned parallel to the flow. In addition, several dysplastic cells were also seen (within white and yellow circles).

Measuring bar: (A-B) 50 µm; (A') 40 µm; (B') 50 µm.

Statistics: n=3 embryos, 6 LVV complexes per genotype.

Fig. S4

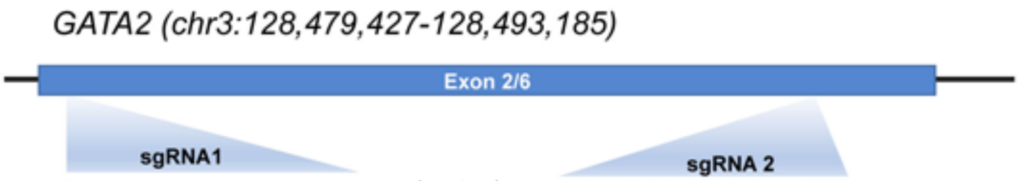


```
AGGGCCGTTGCCGTCTGCACCCAGACCCTGA/190bp/TACTATGCCAACCCCGCTCACGCGCGGGCGCGCGTCT WT       630 reads, 1%
AGGGCCGTTGCC-------------------/190bp/-----------------------GCGCGGGCGCGTCT Δ231   42334 reads, 67%
AGGGCCGTTGCC-------------------/190bp/------------------------CGCGGGCGCGTCT Δ232    7758 reads, 13.2%
AGGGCCGTTG------------AGACCCTGA/190bp/TACTATGCCAACC-------------------CGTCT Δ12,Δ19 2399 reads, 3.8%
AGGGC--------------------------/190bp/-----------------------------------CT Δ248     794 reads, 1.2%
```

Fig. S4: Efficient deletion of GATA2 from HLECs by CRISPR/Cas9.

The second exon (out of six total exons) of *GATA2* is schematically shown. Sequences corresponding to the sgRNAs are in bold and are underlined. The PAM sequences are in red. The ATG of GATA2 is located in between sgRNA1 and sgRNA2. HLEC-2 were infected with lentiviruses expressing Cas9 and the two sgRNAs and selected using puromycin. The region between sgRNA1 and sgRNA2 was PCR amplified and was sequenced using NGS approach. The sequence and contribution of major indels is depicted. These results demonstrate that ~99% of DNA fragments have large deletions in exon 2 of *GATA2*.

Statistics: Sequencing was performed using one batch of selected cells (out of 3 independently selected batches).

Development • Supplementary information

Fig. S5

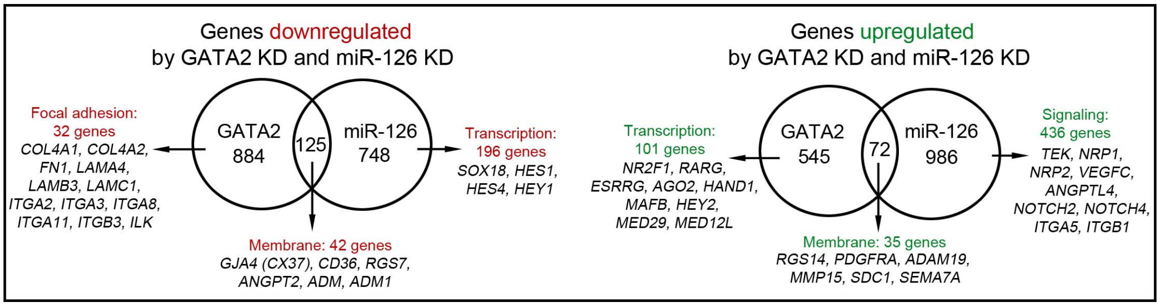

Fig. S5: Selected list of genes regulated by GATA2 and miR-126 in HLECs.

HLECs were treated with shGATA2 or miR-126 sponge and RNA-seq was performed. The differentially regulated genes were analyzed using DAVID.

Table S1: List of genes differentially regulated due to the knockdown of GATA2 or inhibition of miR-126 activity.

Click here to Download Table S1
