# Supplementary material for: GATA2 controls lymphatic endothelial cell junctional integrity and lymphovenous valve morphogenesis through miR-126
Source: Development. 2019 Nov 5;146(21):dev184218. doi: 10.1242/dev.184218 (PMC6857586; doi:10.1242/dev.184218)
Supplement: Reviewer comments [file develop-146-184218_review_history.pdf]

GATA2 controls lymphatic endothelial cell junctional integrity and lymphovenous valve morphogenesis through *miR-126*

Md. Riaj Mahamud, Xin Geng, Yen-Chun Ho, Boksik Cha, Yuenhee Kim, Jing Ma, Lijuan Chen, Greggory Myers, Sally Camper, Debbie Mustacich, Marlys Witte, Dongwon Choi, Young-Kwon Hong, Hong Chen, Gaurav Varshney, James Douglas Engel, Shusheng Wang, Tae-Hoon Kim, Kim-Chew Lim and R. Sathish Srinivasan
DOI: 10.1242/dev.184218

Editor: Benoit Bruneau

Review timeline

Original submission:	23 August 2019
Editorial decision:	23 September 2019
First revision received:	24 September 2019
Accepted:	25 September 2019

Original submission

First decision letter

MS ID#: DEVELOP/2019/184218

MS TITLE: GATA2 controls lymphatic endothelial cell junctional integrity and lymphovenous valve morphogenesis through miR-126

AUTHORS: Md. Riaj Mahamud, Xin Geng, Yen-Chun Ho, Boksik Cha, Yuenhee Kim, Jing Ma, Lijuan Chen, Greggory Myers, Sally Camper, Debbie Mustacich, Marlys Witte, Dongwon Choi, Young-Kwon Hong, Hong Chen, Gaurav Varshney, James Douglas Engel, Shusheng Wang, Tae-Hoon Kim, Kim-Chew Lim, and R. Sathish Srinivasan

I have now received all the referees reports on the above manuscript, and have reached a decision. The referees' comments are appended below, or you can access them online: please go to BenchPress and click on the 'Manuscripts with Decisions' queue in the Author Area.

The overall evaluation is positive and we would like to publish a revised manuscript in Development, provided that the referees' comments can be satisfactorily addressed. Please attend to all of the reviewers' comments in your revised manuscript and detail them in your point-by-point response. If you do not agree with any of their criticisms or suggestions explain clearly why this is so.

Reviewer 1

Advance summary and potential significance to field

In this paper, Mahamud et al report a novel function of Gata2 in maintaining LVV-ECs and VV-ECs during mouse embryonic lymphatic vasculature development. Roles of Gata2 during lymphatic vasculature development have been studied previously using pan-endothelial Cre lines or tamoxifen-inducible Cre lines for deleting Gata2. In this work, the authors used Lyve1-Cre to achieve uniform and constitutive deletion of Gata2 in the lymphatic vasculature. In addition to the

© 2019. Published by The Company of Biologists under the terms of the Creative Commons Attribution License (https://creativecommons.org/licenses/by/4.0/).

blood-filled lymphatic vessel phenotype, mutant embryos also lacked LVs and LVVs. Interestingly, the authors found that the number of LVV-EC was normal in the mutants at E12. However, these LVV-ECs are not aligned perpendicular to blood flow and their morphology appear abnormal. Results from SEM, lineage tracing, and other studies indicate that deletion of Gata2 does not regulate cell proliferation/survival, the differentiation of VV-ECs, but maintains LVV-ECs and VV-ECs and promote their morphogenesis. To gain a mechanistic understanding of the function of Gata2, an RNAseq experiment was carried out using control and Gata2 knockdown HLECs. This led to the identification of several Gata2 targets, including Angpt2, EGFL7 and miR-126. Careful validation analysis reveals that EGFL7 and miR-126 are true targets of Gata2 during the development of lymphatic vasculature both inn vitro and in vitro. Interestingly, a previous study shows that miR126 mutant embryos die in uterus with very severe edema, indicative of defects during the lymphatic vasculature development. Phenotypic characterization of miR126 knockout embryos reveals that the phenotypes of the miR-126 mutants are strikingly similar to that observed in Gata2-LECKO embryos. LVV-ECs were present initially in the mutants at E12 in miR126 mutants. By E16, however, LVVs and VVs were absent in the jugulo-subclavian vein junction in the mutants. These results raised the possibility that some genes involved in later stage of LVVs and VVs development are commonly regulated by Gata2 and miR126. Indeed, in both embryos and HLECs, the expression of VE-Cadherin and Claudin5, two cell junction molecules important for LVs development, are reduced in the absence of Gata2 or miR126. Furthermore, in Gata2 deficient HLECs, overexpression of miR126 rescued the expression of VE-Cadherin and Claudin5. Based on these findings, the authors propose a model in which Gata2-dependent expression of miR126 plays a key role in lymphatic vasculature development by controlling the expression of cell junction molecules such as Claudin5 and VE-Cadherin. Overall, the main conclusions of this paper are convincing and supported by high quality results. The story is complete. The manuscript is generally well written and clearly presented. The finding that the Gata2-miR126-VE-Cadherin/Claudin5 axis is important for maintaining LVV-ECs during lymphatic vasculature is novel and would make an important contribution to the field.

Comments for the author

I would recommend this work for publication if the following points could be addressed.

1) The rationale for showing Spred1 mutant (Fig9) was not well-articulated. It is not entirely clear to the reviewer how these results are linked to the rest of the story and what is the point the authors tried to make by showing these results. If it is not necessary, the authors should consider removing these results from the manuscript.

2) RNA-seq analyses of Gata2 and miR126 knockdown HLECs are important for this work. The authors should do a better job presenting these results. In the case of miR126 transcriptome analysis, the results were simply included in the supplemental table1, together with Gata2 RNAseq data. In its current format, it’s not easy for the reviewer to see and understand these results. The miR126 RNAseq data should be better analyzed bioinformatically. It is also important to show the list of targets genes shared by Gata2 and miR126 in a table. There are 125 shared downregulated genes and 72 shared upregulated genes. Maybe the authors should make a table with the top 20 or 30 from each category and summarize their known functions.

3) Please clarify if HLECs used for Gata2 and miR126 RNAseq experiments were derived from the same human patient. If cells from different patients were used in these experiments, would it be possible that this may be part of the reason why only relatively small number of genes were commonly regulated by Gata2 and miR126?

4) In Fig 7E, contrast of the gel image looks weird. Please use a better picture to replace the one in the paper.

5) Also in Fig7E, please describe how the bar graph on the right was generated. Did the authors normalize the signal from the primers blanking Gata2 binding region to the input, or the “-2Kb” was used for normalization?

© 2019. Published by The Company of Biologists under the terms of the Creative Commons Attribution License (https://creativecommons.org/licenses/by/4.0/).

6) In the Materials and Methods, please provide the sequences of U6 and miR126 primers that were used in qPCR detection.

Reviewer 2

Advance summary and potential significance to field

Authos identified GATA2-miR-126 pathway regulate lymphatic endothelial cell junctional integrity and lymphovenous valve morphogenesis, providing novel mechanistic insights into molecular regulation of lympovenous formation, which important to understanding its associated diseases.

Comments for the author

In this study, Srinivasan et al. used elegant mouse genetics to dissect the role of Gata2 in regulation of lymphatic endothelial cell junctional integrity and lymphovenous valve morphogenesis. They found that GATA2 regulates expression of Claudin5 and VE-Cadherin in lymphatic endothelial cells. They also identified miR-126 as downstream target of GATA2, and miR-126 knockout embryos recapitulate many of the phenotypes of Gata2 knockout mice. Interestingly, over-expression of miR-126 in Gata2 knockout human lymphatic endothelial rescues the expression of cell junctions. This work is interesting and important providing a mechanistic insights of Gata2-miR-126 in regulating lymphatic vascular development. Most of the data presented are of high quality and supporting the conclusion of the work. I support publication of this work and have only a few questions.
1. Authors use Lyve1-Cre for deletion of Gata2. Is this constitutive Cre targeting lymphatic endothelial cell specifically? Authors need to at least comment on this in the Discussion.
2. Is there any specific binding site for GATA2 on the gene locus EGFL7/miR-126? What's the binding motif of GATA2?
3. In miR-126 knockout embryos, is GATA2 expression decreased?
4. Authors used Prox1-CreERT2 also for Gata2 knockout. Does this also target other non-Lymphatic ECs as well?

First revision

Author response to reviewers' comments

Response to reviewers

We thank the reviewers for their positive comments. We provide a point-by-point response to their questions and concerns in the following section. We have highlighted the corresponding changes in the manuscript.

Reviewer 1
Advance summary and potential significance to field
In this paper, Mahamud et al report a novel function of Gata2 in maintaining LVV-ECs and VV-ECs during mouse embryonic lymphatic vasculature development. Roles of Gata2 during lymphatic vasculature development have been studied previously using pan-endothelial Cre lines or tamoxifen-inducible Cre lines for deleting Gata2. In this work, the authors used Lyve1-Cre to achieve uniform and constitutive deletion of Gata2 in the lymphatic vasculature. In addition to the blood-filled lymphatic vessel phenotype, mutant embryos also lacked LVs and LVVs. Interestingly, the authors found that the number of LVV-EC was normal in the mutants at E12. However, these LVV-ECs are not aligned perpendicular to blood flow and their morphology appear abnormal. Results from SEM, lineage tracing, and other studies indicate that deletion of Gata2 does not regulate cell proliferation/survival, the differentiation of VV-ECs, but maintains LVV-ECs and VV-

© 2019. Published by The Company of Biologists under the terms of the Creative Commons Attribution License (https://creativecommons.org/licenses/by/4.0/).

ECs and promote their morphogenesis. To gain a mechanistic understanding of the function of Gata2, an RNAseq experiment was carried out using control and Gata2 knockdown HLECs. This led to the identification of several Gata2 targets, including Angpt2, EGFL7 and miR-126. Careful validation analysis reveals that EGFL7 and miR-126 are true targets of Gata2 during the development of lymphatic vasculature both inn vitro and in vitro. Interestingly, a previous study shows that miR126 mutant embryos die in uterus with very severe edema, indicative of defects during the lymphatic vasculature development. Phenotypic characterization of miR126 knockout embryos reveals that the phenotypes of the miR-126 mutants are strikingly similar to that observed in Gata2-LECKO embryos. LVV-ECs were present initially in the mutants at E12 in miR126 mutants. By E16, however, LVVs and VVs were absent in the jugulo-subclavian vein junction in the mutants. These results raised the possibility that some genes involved in later stage of LVVs and VVs development are commonly regulated by Gata2 and miR126. Indeed, in both embryos and HLECs, the expression of VE-Cadherin and Claudin5, two cell junction molecules important for LVs development, are reduced in the absence of Gata2 or miR126. Furthermore, in Gata2 deficient HLECs, overexpression of miR126 rescued the expression of VE-Cadherin and Claudin5. Based on these findings, the authors propose a model in which Gata2-dependent expression of miR126 plays a key role in lymphatic vasculature development by controlling the expression of cell junction molecules such as Claudin5 and VE-Cadherin. Overall, the main conclusions of this paper are convincing and supported by high quality results. The story is complete. The manuscript is generally well written and clearly presented. The finding that the Gata2-miR126-VE-Cadherin/Claudin5 axis is important for maintaining LVV-ECs during lymphatic vasculature is novel and would make an important contribution to the field.

Response: Thank you for the generous comments.

Reviewer 1 Comments for the author
I would recommend this work for publication if the following points could be addressed.

1)The rationale for showing Spred1 mutant (Fig9) was not well-articulated. It is not entirely clear to the reviewer how these results are linked to the rest of the story and what is the point the authors tried to make by showing these results. If it is not necessary, the authors should consider removing these results from the manuscript.

Response: We agree with the reviewer. Figure 9 is a negative data, which will be suitable for a later study in which the miR-126 targets could be systematically studied. Therefore, we have removed this figure from the manuscript as suggested.

2)RNA-seq analyses of Gata2 and miR126 knockdown HLECs are important for this work. The authors should do a better job presenting these results. In the case of miR126 transcriptome analysis, the results were simply included in the supplemental table1, together with Gata2 RNAseq data. In its current format, it's not easy for the reviewer to see and understand these results. The miR126 RNAseq data should be better analyzed bioinformatically. It is also important to show the list of targets genes shared by Gata2 and miR126 in a table. There are 125 shared downregulated genes and 72 shared upregulated genes. Maybe the authors should make a table with the top 20 or 30 from each category and summarize their known functions.

Response: We agree with the reviewer's concern for clarity. We have generated a new figure (Fig. S5, attached here) in which we have listed some of the genes that are upregulated or downregulated by shGATA2 and miR-126-sponge. We have attempted to select the genes that are known or anticipated to regulate vascular development. I hope this figure is agreeable to the reviewer. We are receptive to any alternative suggestions that he/she may have.

3)Please clarify if HLECs used for Gata2 and miR126 RNA-seq experiments were derived from the same human patient. If cells from different patients were used in these experiments, would it be possible that this may be part of the reason why only relatively small number of genes were commonly regulated by Gata2 and miR126?

Response: Commercially available HLECs from Lonza that were harvested from the foreskin of anonymous donors were used for the first RNA-seq experiment that was performed using shGATA2. HLECs that were generated by co-authors Drs. Young-Kwon Hong and Dongwon Choi were used for

© 2019. Published by The Company of Biologists under the terms of the Creative Commons Attribution License (https://creativecommons.org/licenses/by/4.0/).

the second RNA-seq experiment that was performed using miR-126 sponge (We have modified the Materials and Methods accordingly). The experiments were performed at different times, different lentiviruses were used for the experiment and different kits were used for library preparation. Therefore, differences will certainly exist between the samples. However, we anticipate that GATA2 and miR-126 will regulate the expression of their most physiologically relevant targets irrespective of the LEC-lines that were used.

We do not know why there are limited numbers of commonly regulated genes. Due to limited resources we are unable to repeat all the RNA-seq experiments under identical conditions. However, we have deposited our raw RNA-seq data in Dryad. Our hope is that in the future we (or others) will be able to identify the critical target genes of GATA2/miR-126 axis by comparing our data with similar data that will be generated and deposited by other labs.

4)In Fig 7E, contrast of the gel image looks weird. Please use a better picture to replace the one in the paper.

Response: We have replaced the modified figure with the original un-modified picture. We are also providing an additional figure from an independent experiment here.

5)Also in Fig7E, please describe how the bar graph on the right was generated. Did the authors normalize the signal from the primers blanking Gata2 binding region to the input, or the “-2Kb” was used for normalization?

Response: The graph was generated by calculating the ratio of q-PCR signals generated by using DNA that was immunoprecipitated by anti-GATA2 and IgG antibodies as template. The primers were specific for the GATA2 binding site. We have described it in the Materials and Methods section.

6) In the Materials and Methods, please provide the sequences of U6 and miR126 primers that were used in qPCR detection.

Response: In the previous version of the manuscript we had provided a wrong catlog number for the primers. We have now provided the accurate numbers. However, we do not know the sequence of these primers, as this is Qiagen’s proprietary information.

Reviewer 2

Advance summary and potential significance to field
Authors identified GATA2-miR-126 pathway regulate lymphatic endothelial cell junctional integrity and lymphovenous valve morphogenesis, providing novel mechanistic insights into molecular regulation of lymphovenous formation, which important to understanding its associated diseases.

Reviewer 2 Comments for the author
In this study, Srinivasan et al. used elegant mouse genetics to dissect the role of Gata2 in regulation of lymphatic endothelial cell junctional integrity and lymphovenous valve morphogenesis. They found that GATA2 regulates expression of Claudin5 and VE-Cadherin in lymphatic endothelial cells. They also identified miR-126 as downstream target of GATA2, and miR-126 knockout embryos recapitulate many of the phenotypes of Gata2 knockout mice. Interestingly, over-expression of miR-126 in Gata2 knockout human lymphatic endothelial rescues the expression of cell junctions. This work is interesting and important, providing a mechanistic insights of Gata2-miR-126 in regulating lymphatic vascular development. Most of the data presented are of high quality and supporting the conclusion of the work. I support publication of this work and have only a few questions.

Response: Thank you for the encouraging comments.

1.Authors use Lyve1-Cre for deletion of Gata2. Is this constitutive Cre targeting lymphatic endothelial cell specifically? Authors need to at least comment on this in the Discussion.

© 2019. Published by The Company of Biologists under the terms of the Creative Commons Attribution License (https://creativecommons.org/licenses/by/4.0/).

Response: Lyve1-Cre is constitutively expressed in LECs from as early as E11.5. This Cre line is also expressed in a subset of blood endothelial cells. Please see the data from the lineage tracing experiment that we have performed. Reporter genes (GFP or tdTomato) label most Lyve1+ Prox1+ LECs. In addition, superior vena cava (SVC, a vein) is also labeled.

Accordingly, we have made the following modification to our manuscript (Page 8, lines 6-10).

"Here, we used Lyve1-Cre (Pham et al., 2010) to delete Gata2 (Charles et al., 2006) in the lymphatic vasculature. Using lineage tracing we have determined that Lyve1-Cre efficiently and constitutively labels LECs from E11.5 (data not shown). Lyve1-Cre is also expressed in a subset of blood endothelial cells and leukocytes (Dellinger et al., 2013; Takeda et al., 2016)".

2.Is there any specific binding site for GATA2 on the gene locus EGFL7/miR-126? What's the binding motif of GATA2?

Response: Indeed, Hartmann et al (2016) have reported a GATA2 binding site (GATAA) in the promoter of EGFL7/miR-126. This site is conserved between humans and mice (Figure below). Please note that the GATA2 binding site (underlined in green) is in the complimentary strand.

We have according added the following statement to the manuscript (Page 14, lines 17-19).

"A putative GATA2 binding site (GATAA) is present in the promoter of EGFL7/miR-126. GATA2 associates with this regulatory element in primary human umbilical vein endothelial cells (HUVECs) (Hartmann et al., 2016)".

3.In miR-126 knockout embryos, is GATA2 expression decreased?

Response: We have analyzed the lymphovenous valve region of E15.5 miR-126-/- embryos (Please see figure below). GATA2 is expressed in the few remaining LVV-ECs. This observation together with the RNA-seq data showing that GATA2 expression is not changed in HLECs treated wit miR-126 sponge suggests that miR-126 is not upstream of GATA2.

4.Authors used Prox1-CreERT2 also for Gata2 knockout. Does this also target other non-Lymphatic ECs as well?

Response: We reported this mouse line previously (Srinivasan et al., 2007) and we showed that it is expressed in LECs but not in blood endothelial cells. We have provided a Figure below with a data from a recent lineage tracing experiment. Prox1-CreERT2;R26+/tdTomato embryos were exposed to tamoxifen at E10.5 and analyzed at E14.5. The reviewer could notice that tdTomato is expressed in LECs of the lymph sacs and in the lymphovenous valves. But, very few labeled cells were observed in the superior vena cava (SVC, a vein). Please see a similar data in Figure 3E.

We have according added the following statement to the manuscript (Page 10, lines 4-7).

"To verify the loss of LVV-ECs, we performed lineage tracing using Prox1-CreERT2, which is expressed in the lymphatic vasculature, liver and the lens, but not in blood endothelial cells or blood cells (Srinivasan et al., 2007)".

Second decision letter

MS ID#: DEVELOP/2019/184218

MS TITLE: GATA2 controls lymphatic endothelial cell junctional integrity and lymphovenous valve morphogenesis through miR-126

© 2019. Published by The Company of Biologists under the terms of the Creative Commons Attribution License (https://creativecommons.org/licenses/by/4.0/).

AUTHORS: Md. Riaj Mahamud, Xin Geng, Yen-Chun Ho, Boksik Cha, Yuenhee Kim, Jing Ma, Lijuan Chen, Greggory Myers, Sally Camper, Debbie Mustacich, Marlys Witte, Dongwon Choi, Young-Kwon Hong, Hong Chen, Gaurav Varshney, James Douglas Engel, Shusheng Wang, Tae-Hoon Kim, Kim-Chew Lim, and R. Sathish Srinivasan
ARTICLE TYPE: Research Article

I am happy to tell you that your manuscript has been accepted for publication in Development, pending our standard ethics checks.

© 2019. Published by The Company of Biologists under the terms of the Creative Commons Attribution License (https://creativecommons.org/licenses/by/4.0/).
